# Metagenomics of Atacama Lithobiontic Extremophile Life Unveils Highlights on Fungal Communities, Biogeochemical Cycles and Carbohydrate-Active Enzymes

**DOI:** 10.3390/microorganisms7120619

**Published:** 2019-11-27

**Authors:** Benito Gómez-Silva, Claudia Vilo-Muñoz, Alexandra Galetović, Qunfeng Dong, Hugo G. Castelán-Sánchez, Yordanis Pérez-Llano, María del Rayo Sánchez-Carbente, Sonia Dávila-Ramos, Nohemí Gabriela Cortés-López, Liliana Martínez-Ávila, Alan D. W. Dobson, Ramón Alberto Batista-García

**Affiliations:** 1 Faculty of Health Sciences, Center for Biotechnology and Bioengineering, University of Antofagasta, Antofagasta 1271150, Chile; benito.gomez@uantof.cl (B.G.-S.); claudiavilo@gmail.com (C.V.-M.); alexandra.galetovic@uantof.cl (A.G.); 2 Center for Biomedical Informatics, Department of Medicine, Loyola University of Chicago Stritch School of Medicine, Maywood, IL 90270, USA; qdong@luc.edu; 3 Research Center in Cell Dynamics, Research Institute in Basic and Applied Sciences, Autonomous University of the State of Morelos, Cuernavaca, Morelos 62209, Mexico; hcastelans@gmail.com (H.G.C.-S.); yordanis.perezllano@yahoo.com (Y.P.-L.); sonia.davila@uaem.mx (S.D.-R.); lilimtzavila@gmail.com (L.M.-Á.); 4 Research Center in Biotechnology, Autonomous University of the State of Morelos, Cuernavaca, Morelos 62209, Mexico; maria.sanchez@uaem.mx; 5 Faculty of Zootechnics and Ecology, Autonomous University of Chihuahua, Chihuahua, Chihuahua 31240, Mexico; noga_cl@hotmail.com; 6 School of Microbiology, University College Cork, Cork, Ireland; a.dobson@ucc.ie; 7 Environmental Research Institute, University College Cork, Cork, Ireland

**Keywords:** Atacama Desert, halite, functional metagenomics, endolithic fungi, CAZyme

## Abstract

Halites, which are typically found in various Atacama locations, are evaporitic rocks that are considered as micro-scaled salterns. Both structural and functional metagenomic analyses of halite nodules were performed. Structural analyses indicated that the halite microbiota is mainly composed of NaCl-adapted microorganisms. In addition, halites appear to harbor a limited diversity of fungal families together with a biodiverse collection of protozoa. Functional analysis indicated that the halite microbiome possesses the capacity to make an extensive contribution to carbon, nitrogen, and sulfur cycles, but possess a limited capacity to fix nitrogen. The halite metagenome also contains a vast repertory of carbohydrate active enzymes (CAZY) with glycosyl transferases being the most abundant class present, followed by glycosyl hydrolases (GH). Amylases were also present in high abundance, with GH also being identified. Thus, the halite microbiota is a potential useful source of novel enzymes that could have biotechnological applicability. This is the first metagenomic report of fungi and protozoa as endolithobionts of halite nodules, as well as the first attempt to describe the repertoire of CAZY in this community. In addition, we present a comprehensive functional metagenomic analysis of the metabolic capacities of the halite microbiota, providing evidence for the first time on the sulfur cycle in Atacama halites.

## 1. Introduction

The Atacama Desert, located in Northern Chile, is one of the oldest, arid, and most hostile deserts on Earth. The availability of liquid water is one of the key factors that impose significant restrictions on life [1,2], and as such for many years the Atacama Desert was considered as an uninhabited territory [3]. However, in the last two decades, it has been well documented that soils, sediments, and rocks from this poly-extreme environment harbor a rich microbiota including archaea, bacteria, fungi, protozoa, and algae, as well as viruses [1,4,5,6,7,8,9,10]. Studies on these microbiota have provided important information regarding our perspective of microbial life and microbial community structures and adaptation to poly-extreme environmental conditions [2].

Rocks are probably the nearest-to-optimal habitats that exist in deserts, where they facilitate microbial life. They act as a water reservoir, an exposed surface for water condensation (dew formation), and as natural thermoregulators that confer protection from severe temperature fluctuations. They also serve as solar radiation shields to reduce photodamage derived from the high ultra-violet (UV) radiation index in arid zones [2,11,12,13]. Organisms living in associations within rocks are termed lithobionts, while those living inside of the rocks are classified as endolithic [14].

Halites, commonly known as salt rocks, are evaporitic rocks that contain more than 95% NaCl [2]. They can be considered as poly-extreme habitats since coupled with hypersalinity they are also often exposed to high temperatures, extremely low water activity, and high levels of UV radiation [2]. This type of rock is abundant in Salar Grande, a natural coastal saltern located near the Pacific Ocean in the Southwest of the Tarapacá Region, Chile [2]. As halites are typically colorless or white [15], they are partially translucent to solar irradiation, and consequently photosynthesis can occur in their interiors [2]. As a result of this photosynthetic activity, a carbohydrate-based rich nutrient environment exists within these rocks which is capable of sustaining microbial life. Thus, halites can be considered as micro-scaled salterns that are colonized by a poly-extremophilic microbiota [2,16,17].

Three previous high-throughput-shotgun metagenomic based studies revealed quite low levels of diversity in the endolithic bacterial communities of the Atacama Desert. These analyses reported high levels of NaCl-adapted Bacteria and Archaea (e.g., *Halobacteriaceae* family) and cyanobacteria as the main phototrophs [7,8,18]. These findings confirmed those from a previous study conducted by the Wierzchos group [16,17]. Additionally, a recent study focusing on the microbial community structure in the superficial crusts of halites in the Atacama Desert concluded that prokaryotic communities were largely dominated by *Halobacteriales* and *Bacteroidetes*, together with a few algal and *Cyanobacteria* species [9].

Despite an increase in our overall knowledge of the microbiology of the Atacama Desert, our knowledge on the endolithobiont communities of halites is still quite limited, with little or no metagenomic-based information on eukaryotic microbes, and as a result their biotechnological potential still remains largely underexplored. In addition, there are limited studies describing the diversity and the ecology of fungi in halites [10,19]. Since halite endolithobiont communities represent unique model systems to help increase our understanding of the ecology and physiology of poly-extremophilic microbial consortia [12], further research is clearly required to explore the diversity, ecological roles and biotechnological potential of the microbes living inside these rocks.

We report here on a shotgun metagenomic based analysis on halite nodules from Salar Grande in the Atacama Desert, including both structural- and functional-based approaches. Microbial (archaea, bacteria, fungi, and protozoa) and virus communities were investigated which indicated that the microbiome and virome are typically composed of NaCl-adapted microorganisms and viruses. This is the first in depth metagenomic description of fungi and protozoa as halite endolithobionts. Moreover, a functional analysis of potential biogeochemical cycles involving these endolithobionts provides strong evidence that the halite microbiota has the capacity to make an important contribution to carbon, nitrogen, and sulfur cycles within their ecosystem, but with a somewhat limited capacity to fix atmospheric nitrogen. A structural and functional analyses comparing halite samples collected from different geographical locations in Atacama Desert to metagenomes from desert soils and salterns was also conducted. This analysis indicated that halite microbiomes are more similar to salt adapted microbiomes than to those from other desert ecosystems. Finally, we investigated the repertorire of Carbohydrate-Active enZymes (CAZymes) within the halite metagenome which we found to be particularly enriched in Glycosyl Transferases (GT) and Glycosyl Hydrolases (GH) with potential biotechnological applicability. To date, this is the most comprehensive metagenomic analysis of the microbial communities and their metabolic capacity in Atacama halites.

## 2. Results and Discussion

### 2.1. Taxonomic Classification and Diversity Assessment

Approximately 60% of the assembled contigs from the metagenome were taxonomically classified, which were distributed as follow: 54.63% Archaea, 42.26% Bacteria, and 2.82% Eukaryotes. A small proportion of virus (0.15%) was also detected. To further classify eukaryote reads, sequences were annotated using the Kaiju eukaryote database, resulting in 2.0% of the sequences being assigned to algae, 0.5% to fungi, and 0.32% to protozoa. The relative abundances detected differ slightly from those reported in previous studies. For example, the authors of [8] described a microbiome dominated by Archaea (~71%) followed of Bacteria (~27%) and a small abundance of Eukarya (~1%) in Atacama Desert halite nodules, while a microbiota dominated by Archaea (81%) has also been reported in halite endolithic communities from this Desert [7]. Other studies describing hypolithic microbes in the Namib Desert reported that Bacteria (93%), followed by fungi (5.6%), dominated these communities, with low incidences of Archaea (0.43%) [20]. This suggests that the relative composition of microbial communities that are present in desert rocks may vary and is likely to be influenced by many factors such as the sampling site, rock mineralogy, and its particular climatological conditions, amongst others.

Extreme environments, especially deserts, are particularly challenging for a current taxonomic annotation methodological standpoint [21]. The significant proportion of contigs in our sample (39.35%), with no taxonomic assignment reflects the fact that desert rock microbiomes do not have a close phylogenetic relationship with cultured microbes and viruses for which sequences have been deposited in reference databases. Thus, the microorganisms and viruses colonizing evaporitic NaCl rocks might represent a unique opportunity to identify new genera and species which as yet remain to be characterized, as well as genomes which may encode novel bioproducts and proteins, with potential biotechnological applications.

#### 2.1.1. Fungi as Endolithobionts of Halites

Reports on cultivable fungal communities in the Atacama Desert are quite rare [10,19], which limits our understanding of their potential role within this environment. In addition, there are to our knowledge no culture-independent studies describing the biodiversity of fungi and protozoans in evaporitic rocks. Thus, our knowledge about eukaryote ecology and distribution in these poly-extreme ecosystems is currently quite limited. With this in mind, we attempted for the first time to describe the fungal and protozoan biodiversity of halite nodules by investigated the occurrence of fungi and protozoan sequences within the halite nodule’s metagenome.

**Figure 1:**
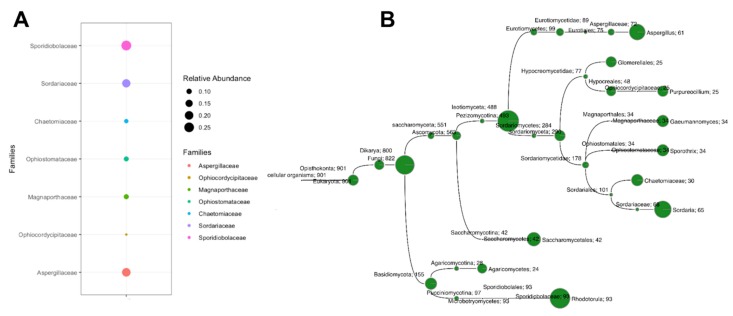
Presence of fungi in Atacama halite metagenome: (**A**) relative abundance of fungal family in the halite metagenome; and (**B**) fungal species annotated as endolithobionts in the halite metagenome.

MEGAN-based taxonomic analysis indicated a limited diversity of fungal families in the halite metagenome, with the *Aspergillaceae*, *Sporidiobolaceae* and *Sordariaceae* families being the most highly represented (Figure 1A). Contigs taxonomically annotated within these three families constituted approximately 65.45% of the total fungal reads. These results indicate that fungi can colonize halites, even though they constitute very high saline environments (NaCl content ~95%). Previous studies have found evidence of the colonization of halites from Yungay, Salar de Llamara, and Salar Grande in the northern Atacama Desert by filamentous fungi [5]. This demonstrates that halites may act as a reservoir for viable fungi. In addition, a limited number of fungal taxa have been isolated from Atacama ecosystems [19], which together with our findings indicate that some fungi can survive and thrive in these environments.

*Rhodotorula*, *Sordaria* and *Aspergillus* (in that order) were the most prevalent (70.19%) of the halite contigs classified at the genus level (Figure 1B). Sequences from *Rhodotorula* represented 100% of those classified in the *Sporidiobolaceae* family (Figure 1B), with most of the sequences showing a high homology with *Rhodotorula graminis* and *Rhodotorula toruloide*. The prevalence of *Rhodotorula* species (typically pigmented basidiomycetous yeasts) has to date been largely described from extreme environments such as glaciers, deep-sea hydrothermal vents, and artic cold deserts [22,23,24]. The recurrent isolation of *Rhodotorula* species from several hypersaline environments clearly demonstrates that this genus is highly ubiquitous and is distributed worldwide.

*Sordaria* contigs were also highly abundant within the fungal community (Figure 1B), resulting in 95.59% of those sequences assigned to the family *Sordariaceae*. Furthermore, some *Sporothrix*- and *Gaeumannomyces*-like contigs were present as members of the subclass *Sordariomycetidae* (Figure 1B), where a large percentage of contigs was assigned to the *Ophiostomataceae* and *Magnaporthaceaer* families. The contigs affiliated with these genera were phylogenetically related to *Sordaria macrospora*, *Sporothrix schenckii* and *Gaeumannomyces tritici*. Interestingly, *S. schenckii* and *G. tritici* are recognized as human and plant pathogens, respectively. However, this is not the first report of pathogenic fungi from arid regions. For example, the human pathogen *Coccidioides* spp. has frequently been isolated from Mexican deserts [25]. In addition, a previous study from rocks in the Atacama Desert reported the presence of some pathogenic *Aspergillus* species [19]. We also detected contigs related to *Purpureocillium lilacinum*, a species of filamentous fungus from the family *Ophiocordycipitaceae* (class *Sordariomycetes*) that has been isolated from deserts and which has demonstrated good potential as a biocontrol agent [26].

Finally, *Aspergillus* was another prevalent genus (Figure 1B) that grouped 84.72% of the contigs affiliated to family *Aspergillaceae*. *Aspergillus fischeri* was the species that showed the highest homology with the halite sequences annotated in this genus. *Aspergillus terreus*, *Aspergillus aculeautus*, *Aspergillus campestris*, *Aspergillus niger*, *Aspergillus glaucus*, and *Aspergillus nomius* were identified as minority aspergilli. The abundance of *Aspergillus* is in agreement with a preliminary culture-based taxonomic report from Yungay halites [27] and a culture-independent approach applied to samples obtained from Llamara ponds [28], both zones being located in the Atacama Desert, where *Aspergillus* was described as the main genus. Aspergilli have also been frequently reported as being dominant in other hypersaline environments and have been described as one of the major genera present in rocks in the Atacama Desert [19]. In general, members of phyla *Ascomycota* and *Basidiomycota* are well represented in our metagenome datasets (Figure 1B). These genera have also been reported in different locations in the Atacama Desert [29,30].

**Figure 2:**
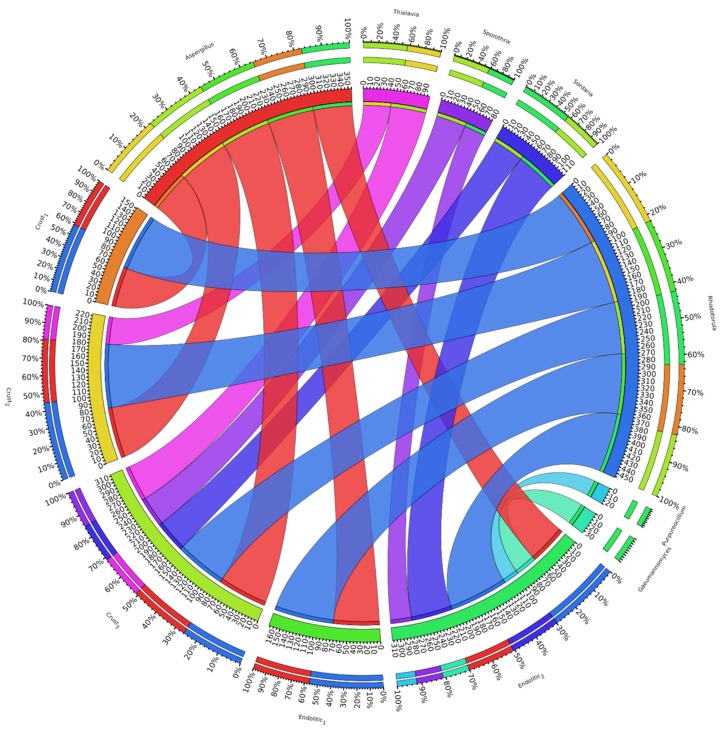
Relative abundance (%) at genus level of fungal communities in both halite crusts and nodules. Crusts 1–3 represent the metagenomes analyzed by Finstad et al [9], while Endolithic 1 corresponds to metagenome analyzed by Crits-Christoph et al [8]. Endolithic 2 represents the halite metagenome analyzed in this work.

With no existing data available with which to strictly compare our results, we performed a taxonomical annotation of genera to classify the fungi present in previously published datasets from halites in the Atacama Desert [8,9] (Figure 2). This analysis revealed that the composition of fungal communities from both halite crusts and nodules is quite similar, with *Rhodotorula* and *Aspergillus* in all cases dominating the communities and sequences from *Thielavia* only being identified in halite crust samples. However, the genera *Sordaria* and *Sporothrix* were largely not represented in the halite metagenomes. When compared with two endolithobiont communities, a higher degree of diversity was observed in our halite metagenome than that which has previously been reported [8].

The fungi detected in our halite metagenome provide a new perspective with respect to our knowledge of the diversity and richness of endolithic fungal communities. Atacama Desert rocks are known to play host to unique fungal communities with diverse lifestyles (e.g., saprobes, parasites, symbionts, human and plant pathogens, epiphytes, endophytes, and mycotoxin producing species) [10,19]. Thus, it is clear that additional detailed sampling focusing on the study of endolithic halite fungi needs to be undertaken, together with additional efforts to elucidate their ecological niches within these rocks and ultimately to explore their potential as a source of novel enzymes and other bioactive compounds.

#### 2.1.2. Protozoan and Green Algae in Halite Nodules

**Figure 3:**
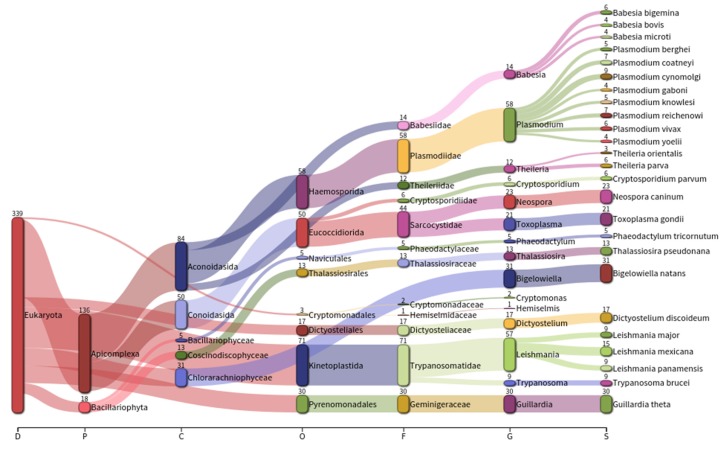
Taxonomic assignment of protozoan in the halite metagenome.

As halophilic protozoa have to date received much less attention than other halophilic microorganisms, their presence in the halite metagenome was also investigated. The protozoa families with the highest representation in halites were *Trypanosomatidae* (20.94%), *Plasmodiidade* (17.11%), and *Sarcocystidae* (12.98%) (Figure 3). Contigs related to 15 protozoa genera were detected with some species previously being described as human pathogens such as *Plasmodium vivax* and *Toxoplasma gondii*. *Plasmodium* and *Leishmania* were the most prevalent genera, with *Plasmodium* species showing the greatest level of biodiversity throughout the protozoa genera present (Figure 3). Protozoa have previously been reported to function in controlling the abundance of prokaryotes and eukaryotes in hypersaline environments [31]. Our findings suggest that halites harbor an interesting and biodiverse collection of protozoa that warrants further investigation, particularly given the deficit in information currently available on free-living protozoa as endolithobionts of halites.

Green Algae from division *Chlorophyta* showed the highest occurrence among the identified eukaryote contigs. Algae have previously been reported in endolithic communities inhabiting halites from the Atacama Desert, with their presence being highly influenced by coastal fogs and the relative humidity of the air [7,8]. This is reflected in the fact that diverse microbial halite communities have been reported in Salar Grande, which is known as a “foggy environment” due to dense coastal fogs which are frequently present in contrast to the very dry Yungay region, from which algae have to date not been reported to be present [7].

#### 2.1.3. Archaea and Bacteria Communities

Although halite-derived prokaryotic populations have been previously analyzed for pooled halite nodules in three previous studies [7,8,18], we also investigated the Archaea and Bacteria profiles in our sample since it is known that the microbial diversity in Atacama locations is highly influenced by environmental conditions at microscale levels [18]. The majority of contigs assigned to Archaea belong to the *Euryarchaeota* phylum (93.75%), which was mainly represented by the *Halobacteria* class (Figure S1). Among Bacteria, 45.25% of contigs were annotated to the *Bacteroidetes* phylum, and in particular to the *Salinibacter* genus, with the *Cyanobacteria* and *Proteobacteria* phyla contributing only 30.56% and 10.18%, respectively (Figure S1). These results are in agreement with those reported by the authors of [7,8,18].

Regarding the *Cyanobacteria* phylum, we found differences with previous studies. In general terms we found a greater overall level of cyanobacterial biodiversity than that which had been reported in previous studies, where only the presence of the *Halothece* genus was described [7,8]. The major genera identified in the metagenome dataset were *Coleodasciculus*, *Halothece*, *Cyanothece*, *Moorea*, *Microcoleus*, *Chroococcidiopsis* (one of the most primitive cyanobacteria known), *Myxosarcina*, *Geitlerinema*, *Micromonas*, and *Gloeocaspa.* Halophilic cyanobacteria from these genera are known to be distributed worldwide and are well adapted to hypersaline habitats where NaCl concentrations can be at levels that are near to saturation (~35%) (e.g., solar lakes, saltern lagoons, and saltern ponds) [32]. Thus, our study shows a diverse community of cyanobacteria that are well adapted to NaCl, which to date had not been observed in halite endolithobiontic samples.

### 2.2. Comparing Halite Microbiomes and Metagenomic Studies in the Atacama Desert

**Figure 4:**
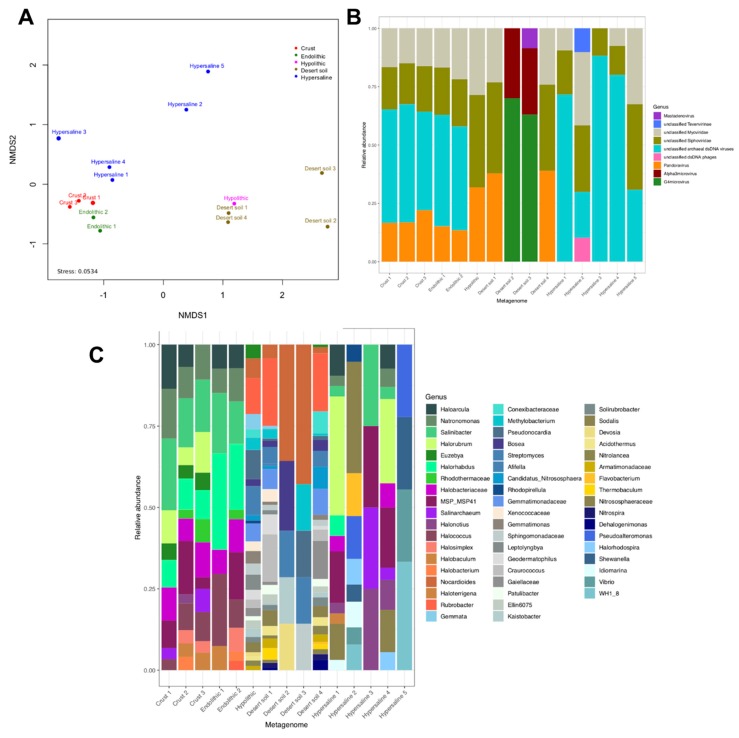
(**A**) Multidimensional scaling analysis based on taxonomic domains. Comparison of our results with previous published metagenome analysis from Atacama´s desert halite rock samples [8,9]. (**B**) Bacteria and Archaea, and (**C**) Viruses. Crusts 1–3 represent the metagenomes analyzed by the authors of [9], while Endolithic 1 corresponds to metagenome analyzed by the authors of [8]. Endolithic 2 represents the halite metagenome analyzed in this work. Metagenome identifies as Hypolithic corresponds with the data obtained by the authors of [22]. Table S1 summarizes the characteristic of metagenomic data identified as Hypersaline 1–5 and Desert soil 1–4.

The multidimensional scaling analysis based on taxonomic domains indicated that the microbial communities inhabiting both the halite crusts and the nodules are consistently clustered (Figure 4A). Some metagenomes from hypersaline environments (salterns) are also closely clustered to the crust and nodule metagenomes, suggesting that the presence of NaCl at concentrations near saturation (~35%) in both halites and salterns may provide a strong constrain for the development of microbial life, thereby restricting ecosystem biodiversity.

To investigate the taxonomic similarity between halite-derived metagenomes with others obtained from similar environments, metagenomic data from desert soils and hypolithic microbial communities from the Namib Desert, which showed close taxonomic relationships in general (Figure 4A), were included in the analysis. It was observed that the halite-derived metagenomes from the Atacama Desert are quite unique and do not share a high degree of overall similarity with microbial profiles from others desert environments, even with hypolithic communities from desert rocks.

Comparison of our results with previous halite-derived metagenomes from Atacama [8,9] reveals that the composition of bacteria, archaea, and viruses is similar in halite crusts and nodules (Figure 4B,C). Interestingly, the hypolithic metagenome from the Namib Desert shows a marked divergence in terms of the prokaryote genera present in the microbial community compared with the halite metagenomes (Figure 4B). While the profile of Bacteria and Archaea appears similar in the halite metagenomes, this composition is markedly different between metagenomes obtained from desert soils and hypersaline environments (Figure 4B). Regarding the viral communities, the halites metagenomes as well as those from the salterns are characterized by a high level of unclassified archaeal dsDNA viruses (Figure 4C). These viruses may represent haloviruses that infect halophilic archaea that are known to be present in high abundance in these extreme ecosystems. Interesting, unclassified archaeal dsDNA viruses were not present in the hypolithic metagenome. On the other hand, *Pandoravirus* appears to be a virus genus that is exclusive to the desert samples as it is absent in metagenomes from salterns (Figure 4C).

The multidimensional scaling analysis indicates that the microbial and viral communities inhabiting halites may represent a unique model with which to investigate microbial life under extreme conditions since they reveal important differences with those present in others related extreme ecosystems (e.g., salterns and desert soils), and even with hypolithic communities from the Namib Desert. In addition, while the metagenomes that we analyzed were obtained from environments which are characterized by low water activity, they are nevertheless associated with specific microbial and viral communities.

Since halite microbiomes are salt-adapted, the biophysical properties of the predicted proteomes have previously been analyzed to determine the molecular adaptations that may take place in response to the ionic conditions present. In this respect, since halophile Archaea are known to maintain their intracellular ionic concentration at a similar level to their extracellular conditions, archaeal proteins are in general acidic and have a low isoelectric point [33,34]. In contract, Bacteria and Eukaryota accumulate compatible intracellular solutes to counteract the high external osmotic pressure and maintain turgor, and therefore only extracellular proteins tend to have lower isoelectric point [34,35,36].

**Figure 5:**
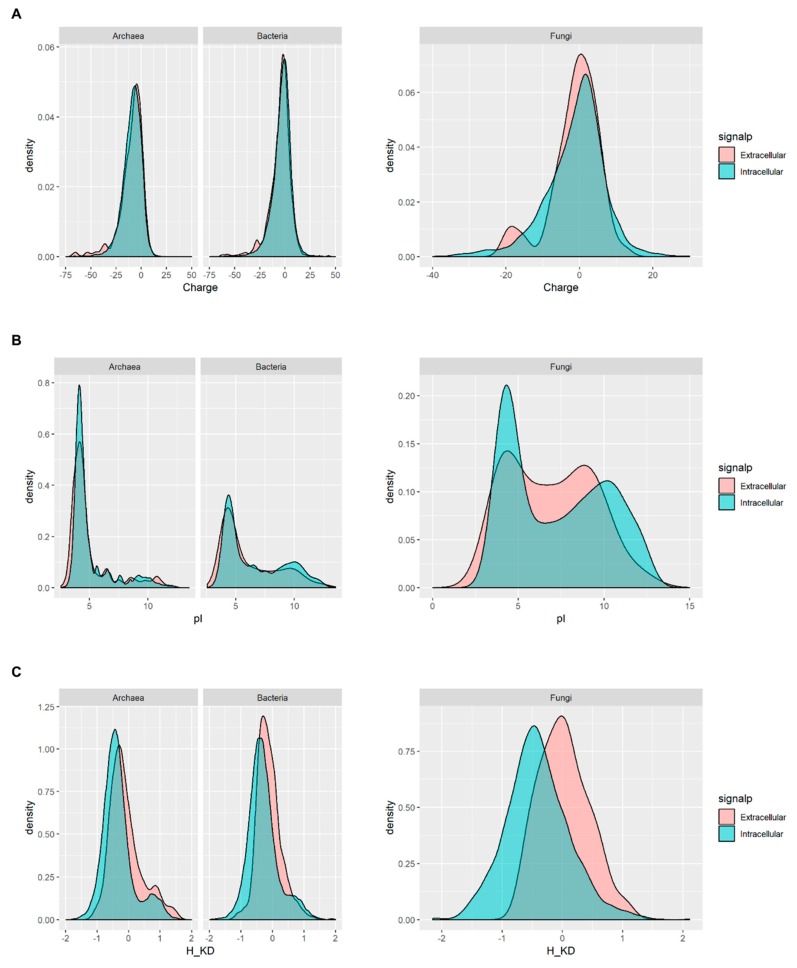
Biophysical properties of the predicted intracellular proteome and predicted secretome (by SignalP v4.0) in the metagenomic sequences from Atacama Desert halites. (**A**) Charge; (**B**) pI; and (**C**) Kyte-Doolittle Hydrophobicity (H_KD) index are represented for Archaea and Bacteria (left) and Fungi (right).

The distribution of charge and pI of the predicted total proteomes in the halite datasets shows that most proteins from Archaea are negatively charged, with a higher proportion having low pIs when compared with either the bacterial or fungal proteins in the halite samples (Figure S2). No significant differences were observed in the distribution of Kyte-Doolittle hydrophobicity index when the total proteomes of the halite microbiota were compared. However, when the secretome and intracellular proteomes of halite microbial communities were analyzed separately, it became evident that, while the overall charge and pI distribution of the predicted fungal secretome does not change significantly (Figure 5A,B), the hydrophobicity of the extracellular fungal proteins is higher than the hydrophobicity of the intracellular proteome (Figure 5C). An increase in the hydrophobicity of extracellular proteins has not previously been reported as an adaptation mechanism in halophilic fungi, although hydrophobic proteins termed hydrophobins (*hfb*) are known to be produced in fungi and an expansion in the number of hydrophobin encoding genes has been reported in halotolerant or in halophilic fungi such as in *Wallemia* species [36,37,38]. However, the increased hydrophobicity of the predicted fungal secretome in our study does not appear to be due to the presence of hydrophobins, as no *hfb* genes were identified in the sample datasets. This suggests the possibility that novel mechanisms of saline adaptation may occur in proteins of eukaryotic origin, but further research would be necessary to confirm this hypothesis.

### 2.3. Functional Description of the Community in Halites

**Figure 6:**
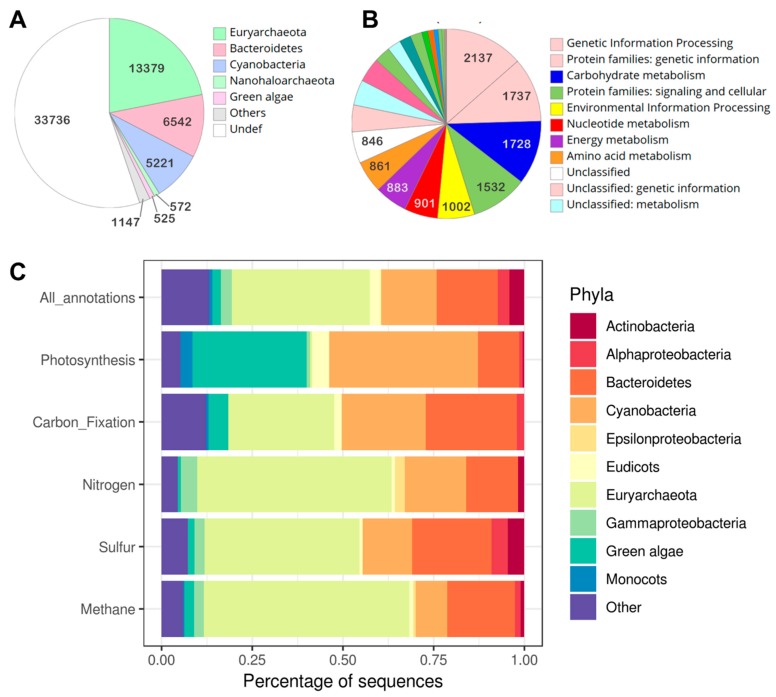
Functional description of the endolithic community in halites: (**A**) open reading frames predicted in different phyla; (**B**) main functional processes enriched in the halite metagenome; and (**C**) the Kyoto Encyclopedia of Genes and Genomes functional annotation.

Shotgun metagenomics can be employed to not only help investigate the composition of the microbial communities that are present in extreme environments, such as halites in the Atacama Desert, but also the functional roles that these microbial community members may be undertaking within these environments. Only 44.8% of the Open Reading Frames (ORFs) predicted from this metagenomic dataset could be functionally annotated (Figure 6A), with many hypothetical or putative proteins of unknown identity being predicted. This indicates that halite microbial endolithic communities may in fact be a reservoir of novel proteins. The main functional processes which appear to be enriched in the halite samples are those related to genetic information processing, carbohydrate metabolism, and cellular signaling (Figure 6B). The Kyoto Encyclopedia of Genes and Genomes (KEGG) functional annotation also indicates that the community is predominantly abundant in the phyla *Euryarchaeota*, *Bacteroidetes*, and *Cyanobacteria*. This is supported by the ORF predictions in Figure 6A, although they appear to be involved in making different contributions to carbon, sulfur, nitrogen, and methane metabolic pathways within the overall community (Figure 6C). This analysis of functional metabolic capacities in halite-derived metagenomes, illuminates for first time the potential contribution of rock-derived metagenomes to sulfur and methane metabolism in the Atacama Desert.

#### 2.3.1. Carbon Cycle

The photosynthetic capacity in the halite microbial community appears to be evenly distributed between *Cyanobacteria* and Green Algae, with genes encoding proteins potentially involved in allophycocyanin and phycocyanin/phycoerythrocyanine antenna complexes (photosystem II) being identified, belonging to the former (Figure S3). In contrast, light-harvesting chlorophyll protein complexes were not present in our functional analysis (Figure S3). This seems to indicate that, although green algae are present, and may contribute to the photosynthetic fixation of carbon, the main role in light harvesting is likely to be fulfilled by *Cyanobacteria*. This is supported by a similar observation from hypolithobiont communities in the Namib Desert [20]. 

The idea that green algae do not appear to be key players in carbon fixation in halite microbial communities is further supported by the observation that sequence composition analysis indicates that these types of fixation pathways are more abundant in the phyla *Euryarchaeota*, *Bacteroidetes*, and *Cyanobacteria*, in that order. This also seems to indicate that autotrophic carbon fixation pathways are likely to be making a considerable contribution towards carbon cycle in this ecosystem.

Nonetheless, it should be considered that differences in relative abundance of biomass and diversity in a sample may result in a bias in the subsequent functional analysis of this sample. For example, all the enzymes involved in carbon fixation pathways associated with photosynthetic microorganisms (e.g., the Calvin-Benson cycle and C4 dicarboxylic acid cycle) were identified in the halite metagenome (Figure S4), which correlates with the high abundance of *Cyanobacteria*. In contrast, we could not find a complete Reductive Acetyl-CoA pathway (or Wood-Ljungdahl cycle), which is proposed to be the closest pathway to a primordial metabolic CO_2_ fixation mechanism and which is widely distributed in *Euryarchaeota* [39]. In particular, the key enzymes involved in this cycle, namely the anaerobic carbon-monoxide dehydrogenase (*acs*A), acetyl-CoA synthase (*acs*B), 5-methyltetrahydrofolate corrinoid/iron sulfur protein methyltransferase (*acs*E), or the NADP+ dependent formate dehydrogenase (*fdh*A), could not be identified in the metagenome dataset (Figure S5). In addition, while some genes encoding enzymes involved in the 3-hydroxypropionate bi-cycle that is present in some green non-sulfur bacteria, in the hydroxypropionate-hydroxybutyrate cycle that occurs in aerobic *Crenarchaeota*, and in the dicarboxylate-hydroxybutyrate cycle that is present in some anaerobic crenarchaeal orders [40] were present, all the genes encoding enzymes involved with these pathways were not present. However while, some of the genera known to be involved with these cycles (e.g., *Sulfolobus*, *Desulfurococcus*, and *Thermoproteus)* were identified in the structural analysis of the community; all the genes encoding enzymes involved with each of these pathways were not present in the metagenome dataset (Figure S5).

We did however identify the main carbon assimilation enzyme involved in the 3-hydroxypropionate bi-cycle, namely the acetyl-CoA carboxylase that catalyzes the conversion of acetyl-CoA to malonyl-CoA [40]. Regarding the hydroxypropionate-hydroxybutyrate cycle, the propionyl-CoA is typically carboxylated to *(S)*-methylmalonyl-CoA (main reaction) by the propionyl-CoA carboxylase and it has been reported that this enzyme is similar or identical to acetyl-CoA carboxylase [41]. In addition considering that the enzymes involved in succinyl-CoA production was also present (Figure S5), our results strongly suggest that these two metabolic cycles for CO_2_ fixation may potentially occur within the halite microbial community particularly given that the enzymes which catalyze the main two metabolic reactions for carbon assimilation have been identified in the metagenomics datasets. Also, given that very few enzymes involved in these CO_2_ fixation cycles have to date been identified in many prokaryote genomes [41], it is likely to be the reason so few of these enzymes were annotated in this halite metagenome.

Another very interesting finding is that the genes encoding enzymes involved in the Reductive citrate cycle (also known as the Reverse Krebs cycle, Reverse tricarboxylic acid cycle or Reductive Arnon-Buchanan pathway) found in *Proteobacteria*, green sulfur bacteria, and microaerophilic bacteria of the early bacterial phylum *Aquificae* [39,42] were nearly completely present in their entirety in the metagenomics datasets. This Reductive citrate cycle allows the synthesis of carbon compounds from CO_2_ and water, and has been proposed as an alternative to inorganic carbon fixation in the reductive pentose phosphate cycle that occurs in *Cyanobacteria* [39]. Only the genes encoding citryl-CoA synthetase (*ccs*A), the citryl-CoA lyase (*ccl*), and the ATP citrate (pro-S)-lyase (*acly*) that catalyze the conversion of citrate to oxaloacetate could not be identified in these datasets. The possibility exists however that oxaloacetate could be formed via pyruvate and thus that the enzymes involved in this conversion are entirely present in the halite metagenome (Figure S5).

In addition, some bacteria such as *Heliobacteria* for example are known to possess an incomplete, yet functional Reverse Krebs cycle [40]. The high number of proteobacterial sequences—confirming the presence of this phylum in the community—suggests that the Reductive citrate cycle is likely to be functional in halites. Interestingly, the Reverse Krebs cycle has also been proposed as a pathway which may have been involved in the formation of prebiotic early Earth and consequently may be of significance from the perspective of the origins of life [43].

In addition, it has also been recently demonstrated that more than half of the Reverse Krebs cycle, which functions as an anabolic pathway and is central to carbon metabolism, can be photochemically activated in the presence of certain metals (e.g., iron, zinc, and chromium), ensuring completion of the cycle in a non-enzymatic fashion. This supports the potential feasibility of this pathway being involved in a primitive form of carbon anabolism in an acidic, metal-rich reducing environment [44]. Thus, it is clear that desert rocks may be of particular interest from an astrobiology perspective, given that they can mimic abiotic extraterrestrial environments.

In a previous halite endolithic metagenome analysis, the Calvin-Benson cycle was identified as the only metabolic pathway likely to be involved in CO_2_ fixation [8]. Our results indicate that other autotrophic carbon fixation pathways (e.g., Reverse Krebs cycle, 3-hydroxypropionate bi-cycle, hydroxypropionate-hydroxybutyrate cycle, and dicarboxylate-hydroxybutyrate cycle) may also be used by endolithic microbial communities and that carbon fixation metabolism in halites may be a more complex process than previously believed. Further research will be required to determine whether the community maintains a low oxygen tension, thereby favoring anaerobic carbon fixation by *Proteobacteria*; maintains conditions favoring the enrichment of *Cyanobacteria*, thereby producing an aerobic environment; or whether both members co-inhabit the substrate in different layers, thereby ensuring appropriate conditions for each to thrive.

**Figure 7:**
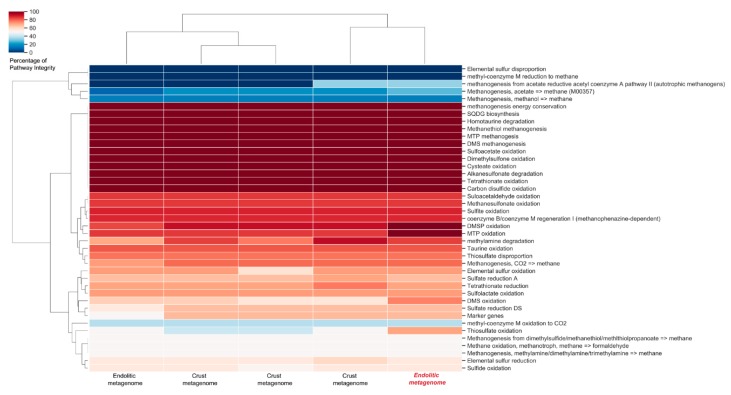
Clustering hierarchical of pathways of the biogeochemical cycles in halites. Hierarchical heat map is shown where the most pathways in biogeochemical cycles are marked in red colors. Crusts 1–3 represent the metagenomes analyzed by the authors of [9], while Endolithic 1 corresponds to metagenome analyzed by the authors of [8]. Endolithic 2 represents the halite metagenome analyzed in this work.

Another important part of the biogeochemical carbon cycle is the production and consumption of environmental methane [45,46]. *Euryarchaeota* were until recently considered the archaeal origin of methane metabolism, by playing a major role in the carbon cycle through either the formation or oxidation of methane. Although more primitive *Archaea* are now identified as methanogens and methanotrophs, members of the *Euryarchaeota* genera are still considered the main contributors to methane cycle globally [46]. In the halite community, genes associated with methane metabolic pathways were mainly classified as belonging to *Euryarchaeota* and to a lesser extent into *Bacteroidetes* and *Cyanobacteria*. However, we could not identify the key enzyme marker for archaeal methane metabolism, e.g., the methyl-coenzyme M reductase complex (*mcr*) (Figure S5). Interestingly, this marker was also not identified in previous metagenome studies of communities thriving inside [8] or at the crust of halites [9] (Figure 7). Nonetheless, our structural analysis indicated that several methanogenic genera (<1%) can be identified in the community, namely *Methanosarcina*, *Methanothrix*, *Methanobrevibacter*, and *Methanobacterium*, among.

#### 2.3.2. Nitrogen Metabolism

The nitrogen cycling strategy that microbial communities typically implement is Assimilatory Nitrate Reduction to Ammonia (ANRA), which is commonly referred to as ammonification and is performed by both bacteria and fungi [47,48]. The ANRA pathway involves internalization of nitrate/nitrite by means of Major Facilitation Superfamily (MFS) type membrane transporters, conversion of nitrate to nitrite by ferredoxin-nitrate reductase (*nar*B) and from nitrite to ammonia by the ferredoxin-nitrite reductase (*nir*A) (Figure S6). This respiratory process is generally anoxic in both bacteria and fungi [49]. The ammonium produced by this pathway is further assimilated by the activity of glutamine synthetase (*gln*A) through the conversion of glutamate to glutamine [48]. Genes encoding enzymes involved in nitrification reactions that convert ammonium back to nitrite/nitrate under aerobic conditions were not identified in our analysis (Figure S6), supporting the idea that the ANRA pathway is potentially the only mechanism of nitrogen cycling in the halite community, and that it is most likely acting under anaerobic or microaerophilic conditions [47].

In addition, *nif* genes were found not to be present in the halite community in this study (Figure S6). This is in agreement with previous reports from Atacama Desert halites, which concluded that there is little or no evidence of nitrogen fixation metabolism in crusts [9] or in endolithic communities [8]. Studies conducted in hyper-arid deserts in China describing hypolithic microbial communities have also reported the absent of prokaryote phylotypes related to nitrogen fixation with *nif* genes not being detected in PCRs using *nif*H specific primers [49]. In contrast, earlier studies described the presence of bacteria in Atacama soil samples that were capable of nitrogen fixation [50]. Supporting this observation, acetylene reduction tests have demonstrated that hypolithic communities can potentially fix atmospheric nitrogen in cold deserts [51]. However, a hypolith metagenome sequence database from the Namib Desert reported low levels of *nif*H genes, indicating a decreased potential for this hypolithobiont community to undertake nitrification processes [20]. Thus, this metagenomic study supports the earlier hypothesis of the authors of [9] indicating that the biotransformation of environmental nitrate present in Atacama halites is the main source of metabolically utilizable nitrogen for the halite microbial communities.

#### 2.3.3. Sulfur Metabolism

The common markers of Dissimilatory Sulfate Reduction and Oxidation (DSRO) (e.g., *apr*A and *apr*B, the adenylylsulfate reductase genes, and *dsr*A and *dsr*B, the dissimilatory sulfite reductase genes) were not identified in the metagenomic contigs (Figure S7). This result is supported by the structural analysis given that contigs related to anaerobic sulfate reducing bacteria (e.g., *Desulfovibrio*, *Desulfomonile*, *Desulfolobus*, *Desulfohalobium*, *Desulfonatronospira*, *Desulfomaculum*, *Desulfomicrobium*, *Desulfofustis*, *Desulfococcus*, and *Desulfobacterium*) were detected at very low levels (<1%) in our halite metagenome. The absence of sulfate-reducing microorganisms could be an ecological strategy, as the metal sulfides that they are likely to produce would darken the rock substrate and prevent light penetration, thus potentially impairing photosynthesis. A previous metagenomic dataset from Namib Desert also reported the absent of genes for dissimilatory sulfate reduction and sulfide oxidation [20].

In contrast, genes encoding enzymes involved in all the components of the Assimilatory Sulfate Reduction (ASR) pathway were identified, thereby indicating the likely assimilation of sulfur for cellular metabolism within the microbiome (Figure S7). Sulfate is one of the major chemical components which is transported by the coastal fog to the Salar Grande region [50] and is used by the microbial communities as their main source of sulfur. All the genes involved in the assimilation of sulfide into pathways involved in amino acid metabolism (e.g., L-serine, L-cysteine, L-methionine, and L-homocysteine) were found in the metagenome (Figure S7).

The Sulfur-OXiding (SOX) system, composed of the SoxA, SoxYZ, SoxB, and SoxCD proteins, is known to be involved in the conversion of thiosulfate to sulfate and is employed by many facultative chemolithotrophic *Proteobacteria*. We identified the s*ox*X (thiosulfate dehydrogenase) and s*ox*C (cytochrome *c* oxidoreductase) genes, two of the main genes encoding proteins involved in this oxidative process, in the halite metagenome [52]. It has been reported that the *sox* gene cluster may be partially incomplete in some bacteria that carry out sulfur-oxidation of the thiosulfate molecule [52]. Supporting this, the s*ox*YZ complex is known not to be an intermediate of the SOX pathway in the bacterium *Paracoccus pantrophus* [53]. Indeed, only s*ox*B, a gene encoding for thiosulfohydrolase activity, is missing in the halite metagenomic dataset (Figure S7). This function could however be carried out by other thiosulfohydrolase-like proteins within the halite microbiome [52]. However, the *sox*B gene has been described as a crucial marker gene for the SOX system in environmental samples [54]. In contrast with our results, Vikram and coworkers detected *sox*B genes in a metagenome derived from the hypolithobiont in the Namib Desert [20]. Nonetheless, it appears likely that sulfate can be produced from thiosulfate within the halite metagenome. Thus, our sulfur cycle analysis indicates that the halite microbial consortia show a limited genetic repository likely to be involved in sulfate oxidation.

### 2.4. Biogeochemical Cycles in Halites: A Comparison

An analysis including all the halite-derived publicly available metagenomics datasets (Figure 7), indicated that the profile of pathways involved in biogeochemical cycles is very similar in all these datasets. A pathway integrity analysis showed, as previously reported, that the methanogenesis and DSRO pathways are not complete in halite samples. In addition, no genes potentially encoding enzymes involved in complex nitrogen cycling can be identified in any of the microbiome studies of the halite communities, other than those involved in the ANRA pathway. Taken together, these results indicate similar community functionality, both for endolithic and epilithic halite communities, as well as in samples from different Atacama geographical locations. It also suggests that the microbial crust halites communities appear to favor micro-anaerobic niches, given the metabolic and microbial profiles which have been observed, and particularly given that many of the enzymes potentially involved are likely to be sensitive to oxygen.

Even though there has been increased interest in studying the microbial communities that colonize rocks in poly-extreme deserts, the contribution that these microbes make to the biogeochemical cycles in this ecosystem is as yet not fully understood. This is the first detailed shotgun metagenomics study that describes a functional comparison of biogeochemical cycles in halite samples collected from different geographical locations in the Atacama Desert. This analysis suggests that these halite communities possess the capacity to make an important contribution to carbon, nitrogen, and sulfur cycles, but possess a limited capacity to fix nitrogen. Further studies (e.g., meta-transcriptomic and meta-proteomic approaches) will be required to further elucidate the precise ecological niches of the microbial populations within the overall community that are involved in these different biogeochemical cycles. This will need to be coupled with additional metagenomics approaches involving deep sequencing to help increase our understanding of the functional microbial networks that are functioning in these biogeochemical cycles, with a particular emphasis on under-represented members of these communities.

### 2.5. Prediction and Analysis of Carbohydrate-Active Enzymes from Halites

**Figure 8:**
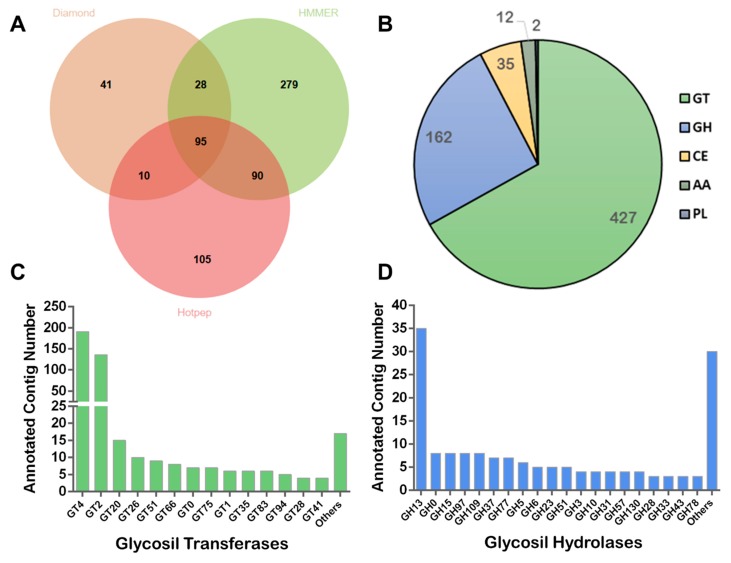
Prediction of CAZymes in the halite metagenome: (**A**) CAZyme composition using three different algorithms; (**B**) CAZyme annotated as Glycosyl Transferases (GT), Glycosyl Hydrolases (GH), Carbohydrate Esterases (CE), Auxiliary Activities (AA), and Polysaccharide Lyases (PL); and (**C**,**D**) family distributions of GT and GH, respectively.

Three different algorithms, namely Diamond, HMMER and Hotpep, were used to analyze our metagenomics dataset for the presence of CAZy genes. CAZy enzymes typically catalyze the breakdown, biosynthesis, or modification of carbohydrates. In addition, they transfer carbohydrate moieties to different acceptor molecules, including proteins, lipids, nucleic acids, secondary metabolites, and other oligo- or polysaccharides [55,56]. Their action within microbial communities typically results in the degradation of various substrates and the production of specific polymers and sugar-modified proteins or metabolites. There has to date been little interest in documenting the presence of CAZy genes within halite samples, which is perhaps surprising given the important role that these enzymes are likely to play in shaping the overall structure and dynamics of the community. Our analysis indicated the presence of a vast repertoire of CAZymes within the halite metagenome (Figure 8A), with GT being the most abundant class present, followed by GH (Figure 8B).

In total ,427 putative GT genes belonging to 25 different families were identified within the halite metagenome. Among the GT, there was a distinctive enrichment in the GT4 and GT2 classes, with a more than ten-fold higher levels of annotated protein in these two classes than in other individual families (Figure 8C). This enrichment reflects the predominance of Archaea in the community, given that they only contain these two classes of GT enzymes [55]. GT4 is a family of enzymes that can use both nucleotide- and phospho-sugar donor molecules, and with most family members being involved in central metabolic pathways [55]; therefore, it is not surprising that a high number of these are present within the halite microbial communities. On the other hand, GT2 group members are typically involved in the synthesis and modification of long polysaccharide structures such as cellulose, chitin, glucans, and hyaluronan, amongst others [55]. These proteins could therefore be informative with respect to the overall community structure, as they are involved in the synthesis of the distinctive outer layers (e.g., cell wall and polysaccharide layer) of many microorganisms. GT2 group members might also be involved in microbial adaptations to salinity, as it has been observed that halophiles enlarge or modify their cell walls allowing them to endure sustained external insults [32,34,36].

Thirty five CE genes and other 12 genes encoding for AA—redox enzymes with synergic activities with CAZyme members—were also identified in the halite metagenome (Figure 8B). CE10 and CE4 families were the best represented with 40% and 23% of the annotated CE sequences, respectively. While CE10 includes esterases acting on non-carbohydrate substrates, CE4 members comprise esterases with activity on peptidoglycan, chitin, and xylan (http://www.cazy.org).

In total, 162 putative GH genes belonging to 42 different families were also identified within the halite metagenome (Figure 8D). GH are the hydrolytic machinery that enable the degradation of molecules with glycoside bonds, and thus determine whether a microorganism can use a particular substrate as a carbon source. Among the GH, there was a marked enrichment in the GH13 family (21.6%) (Figure 8D). In addition, members of different GH families of potential biotechnological interest were also identified (e.g., GH3, GH5, GH6, GH10, GH43, and GH51).

The GH13 family is known as the α-amylase family, as its members can hydrolyze the α-glycosidic bonds characteristic of amylose, present in storage polymers such as starch and glycogen [57]. It is not surprising that this amylase family is represented to such a large extent in the halite metagenomics samples since the GH13 family is predominantly comprised of archaeal amylopullulanases, similar to α-amylases [57]. Amylopullulanases are bifunctional enzymes with the capacity to hydrolase α-1-4- and α-1-6-glycosidic linkages and degrade a huge range of carbohydrate such as amylose, pullulan, amylopectin, and related oligosaccharides [57]. Moreover, it has also been reported that some halophilic *Euryarchaeota* possess amylases belonging to a new GH13 subfamily [57].

**Figure 9:**
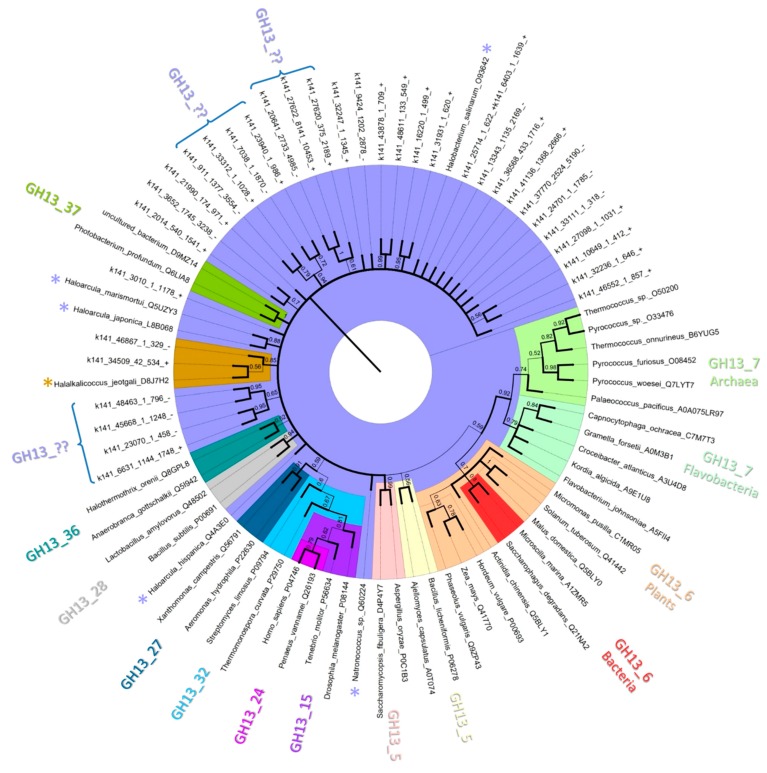
Phylogenetic tree of the family GH13 α-amylases. The phylogeny was prepared based on the alignments of seven conserved sequence regions. Sequences from different GH13 subfamilies were considered in the analysis and are identified by their UnitProt Accession Numbers and source names.

Because of the high incidence of GH13 in the halite metagenome, we investigated their phylogenetic relationships with some type-sequences of different GH13 subfamilies. Currently, GH13-7 is the subfamily that mainly groups the α-amylases from halophilic Archaea [58]. An evolutionary tree based on the Maximum Likelihood method revealed that many GH13 α-amylases identified from the Atacama halites display a distant phylogenetic relationship with those well characterized, even with those sequences classified in the subfamily GH13-7 (Figure 9). At least three novel GH13 α-amylases subfamilies could be distinguished from the phylogenetic tree with clusters that are unrelated to typical GH13 subfamilies (e.g., GH13-7 from *Pyrococcus* spp. and *Thermococcus* spp.) (Figure 9). The α-amylases that were identified in the halite metagenome do not contain the QPDLN amino acid signature typically found in the Conserved Sequence Region (CSR-V) of the canonical GH13-7 amylases. This is supported by the phylogenetic analysis as no direct evolutionary relationship appears to exist with amylase members belonging to GH13-7 (Figure 9). In addition, several halite GH13 sequences formed solitary branches, reflecting the presence of other novel GH13 members (Figure 9). Only two halite amylase sequences share a phylogenetic branch with the α-amylase of *Halalkalicoccus jeogtali* (Figure 9), which remains is currently not classification at the subfamily level [57].

These findings suggest that Atacama halites could be a novel source of halophilic GH13 α-amylases, but further in silico and detailed biochemical characterization would be required to formally proposed these as new GH13 subfamilies. However, a few of these α-amylases are distantly clustered with the GH13-7 subfamily, which have been described as potential α-amylases.

Due to the increased recent interest in GH due to their potential in biorefinery applications, we decided to further analyze these GH that had been annotated as putative endoglucanase. Seventeen GH sequences annotated from the halite metagenome and potentially involved in the degradation of carbohydrates were modeled using i-TASSER (Table 1). 3D-models were associated to a GH family based on the templates and structural analogs, using crystalized proteins deposited in the Protein Data Bank (PDB) as a reference. In total, 10 GH families were identified in the set of 17 halite sequences. Models displayed C-score values ranging 0.03–0.30, which were calculated based on the significance of threading template alignments [59]. C-scores values can be assigned in the range [–5, 2] where a higher value reflects a model with a high level of confidence [59]. The confidence of 3D-models can also be estimated by TM values (0, 1], which were calculated to be from 0.60 to 0.89, where TM = 1 indicates a perfect structural similarity between two protein structures for a modeling round [59]. According to the values obtained, it was clear that the majority of the 3D models constructed by i-TASSER were of a high quality with a few exceptions with models that showed poor quality based on C-score, TM-score, and Root-Mean-Square Deviation (RMSD) of atomic position values. The modeled proteins displayed homology with xylanases, arabinofuranosidases, endo-glycosidases, endo-1,4-β-mannanases, endo-1,6-β-glucanase, β-1,3-glucanase, β-glucosidase and acetylglucosaminidase, amongst others (Table 1). Thus, this 3D analysis demonstrates that the microbial communities of Atacama halites harbor a large diversity of genes potentially involved in carbohydrate degradation.

Although the enzymatic activities of GH genes (e.g., GH13 and the 3D modeled GH) analyzed here have not yet been biochemically evaluated, the data obtained strongly suggests that microbial communities inhabiting evaporitic rocks such as halites possess an interesting repertoire of GH proteins that should be further investigated for utility in potential biotechnological based industrial biorefineries applications.

## 3. Materials and Methods

### 3.1. Sampling, Metagenomic DNA Extraction, and Sequencing

Six halite rocks with an average size of 20 cm × 25 cm and an average weight of 1–3 kg were collected (20°56.310′ S; 70°00.319′ W) from Salar Grande into sterile plastic bags (Nasco, Whirl-Pak) (Figure S8). The rocks were sampled within a 20 m^2^ area and sampling was conducted during spring (October 2013). The halites were fractured into large pieces under aseptic conditions. All the halites showed endolithic microbial colonization, and after fracturing the colonization zone (identified as a greenish powder inside the rocks) was collected (Figure S8). DNA extraction was conducted on each of the six halites using the PowerSoil DNA Isolation kit (MoBio Laboratories Inc., Solana Beach, CA, USA) and was subsequently pooled. The sampling strategy was as previously described [7,8], with five halite nodules being pooled, in duplicate, for DNA extraction. The quality of the extracted metagenomic DNA was evaluated on 1% agarose gels and quantified spectrophotometrically at 260 nm. Shotgun sequencing was conducted on the Illumina HiSeq2500 platform in the Center for Biomedical Informatics at Loyola University of Chicago, Maywood, IL (USA). The reads obtained had an average length of 300 bp, producing a total of 3,690,128 reads.

### 3.2. Sequence Quality Control, and Assembly

Firstly, a sequence Quality Control (QC) analysis was performed using FastQC software version 0.11.8 (https://www.bioinformatics.babraham.ac.uk/projects/fastqc/) where the data had an average quality score of 30 which corresponds to a 0.001 error rate. Later, Trimmomatic (software version 0.38, http://gensoft.pasteur.fr/docs/Trimmomatic/0.38) was used to remove adapters and low-quality reads [60]. Duplicated (overrepresented) sequences were eliminated using CD-HIT-dup (software version 4.6.8) (–e 0.003 -m f) [60]. Trimmed reads were assembled and contigs were obtained using MegaHit software version 1.1.1-2 (https://github.com/voutcn/megahit). Briefly, an assembly algorithm based on Bruijn graphs with paired-end mode, k min = 21, k max = 131 and k step = 10 was used [61].

### 3.3. Taxonomic Classification and Functional Analysis

Taxonomic annotation of Archaea and Bacteria was performed using Kaiju, a novel metagenomics pipeline that allows the classification of up to 10 times more contigs from environmental metagenomes with high sensibility and precision [21]. This algorithm translates all the metagenomics sequences into all six possible reading frames. Since protein sequences are more evolutionarily conserved, using the maximum exact matches for each amino acid sequence in reference databases, Kaiju assigns a taxonomic identifier according to the corresponding taxon. To achieve this, Kaiju employs the Burrows–Wheeler transform algorithm, which shows greater precision in the correct taxonomic classification than methods based on the *k*-mer distribution [21]. The taxonomic classification was visualized using Pavian [62].

The classification of viruses and fungi was conducted with the standalone Nucleotide Basic Local Alignment Search Tool (BLASTn) against in-house databases using the following parameters: e-value = 0.0001 and word size = 9 [63]. Firstly, two local databases containing complete or draft viral and fungal genomes deposited in the National Center for Biotechnological Information (NCBI) were constructed. For the taxonomic assignation of viruses and fungi, all the assembled metagenomic sequences were blasted against these local databases using the aforementioned parameters and a taxonomic classification based on the Lowest Common Ancestor (LCA) was performed using MEGAN (software version 5.10.6, https://computomics.com/megan.html). For this analysis, the set of the best twenty scoring BLASTn hits were parsed and the taxonomic classification performed using the following parameters: min support = 1, min score = 70, identity = 50, and top percent = 10 [64]. Relative abundance tables were obtained from MEGAN software, and stacked graphs were constructed using the ggplot2 library within the R language environment.

For the functional analysis of metagenomic contigs, the prediction of coding sequences was carried out using FragGeneScan [65]. A functional annotation was performed with the amino acid sequences using the SuperFocus program against the SEED database clustered to 90% sequence similarity (db90) and using default parameters [66]. Further functional annotation was carried out using the GhostKOALA tool [67] from the KEGG database, allowing the mapping of metagenomic sequences to pathways and biological process KEGG modules. Biophysical properties (i.e., charge, pI, Kyte-Doolittle Hydrophobicity index) of the predicted proteome were analyzed across Kingdoms in all the metagenomic sequences using the R Package Peptides (https://github.com/dosorio/Peptides/). The potential secretome was identified by SignalP v4.0 (http://www.cbs.dtu.dk/services/SignalP-4.0/), and the biophysical properties of extracellular and intracellular proteins were compared.

### 3.4. Comparison of Halite-Derived Microbiomes of the Atacama Desert

A comparison was conducted using currently available metagenomic data, e.g., the metagenomic sequences from halite-derived communities obtained in [8,9]. Additionally, metagenomics data obtained from desert soils, hypersaline environments (salterns) and hypolithic microbial communities from the Namib Desert were also used (Table S1). All these metagenomes were processed with the same pipeline that was used for our metagenomics sample and compared to evaluate their taxonomic similarity. The comparison was performed in R using Vegan library via a multidimensional scaling procedure considering taxonomic domains. Moreover, a biogeochemical cycle analysis was performed through identification of the completeness of metabolic pathways involved in carbon, methane, nitrogen, and sulfur cycling, using Multigenomic Entropy Based Score (MEBS) software [68].

### 3.5. CAZymes Annotation and Bioprospection

CAZy enzymes were identified by the annotation of CDS sequences using the dbCAN2 tool [69] with all the default search algorithms implemented in that software. Functional annotation was obtained for GH, GT, Polysaccharide Lyases (PL), Carbohydrate Esterases (CE), and Auxiliary Activities (AA) when one of the three-search/prediction algorithms (HMMER, DIAMOND, and Hotpep) predicted a CAZY family.

For the phylogenetic characterization of GH13 sequences, the predicted GH13 sequences from the metagenome were aligned to the previously described GH13 Multiple Sequence Alignment (MSA) profile [58]. The MSA was carried out in MEGA [70] using the MUSCLE algorithm with default parameters [71], with the resulting MSA being manually modified in AliView [72] to remove unwanted sequence blocks. For phylogenetic reconstruction, the best substitution model was selected in MEGA based on the lowest BIC scores (Bayesian Information Criterion). Phylogenetic distances were calculated using the LG model [73] assuming gamma distributed evolutionary rates among sites and evolutionarily invariable sites. The Maximum Likelihood Tree was performed with the Bootstrap method using 1000 iterations. The final consensus tree was further modified for visualization using FigTree (http://tree.bio.ed.ac.uk/software/figtree/).

Seventeen sequences of the GH families potentially involved in lignocellulose degradation were selected and modeled. 3D modeling rounds with no restrictions were made using the i-TASSER server (https://zhanglab.ccmb.med.umich.edu/I-TASSER/) [59] and a structure model was obtained for each GH sequence.

### 3.6. Data Availability

Sequence data were deposited in the NCBI Sequence Read Archive under the BioProject Accession Number PRJNA540872.

## 4. Conclusions

Studies on the microbiome of extreme environments such as halite nodules from the Atacama Desert are important in helping to increase our understanding of microbial community structures that are present and how they have adapted to poly-extreme environmental conditions. Structural analysis of the community indicated that, while the halites appear to harbor an interesting and biodiverse collection of protozoa, they possess quite a limited diversity of fungi. Functional analysis indicated that the endolithobionts make an extensive contribution to biogeochemical cycles involving carbon, nitrogen, and sulfur, but possess a limited capacity to fix nitrogen. Finally, the halite metagenome contains a large number of CAZymes including GT and GH, amylases, and glycoside hydrolases, making this ecosystem a potentially interesting source of novel enzymes with biotechnological potential.

## Figures and Tables

**Table 1:** Statistics of the 3D modeling of glycoside hydrolases identified in halite metagenome.

Sequence ID	iTASSER ID	C-Score	TM-Score	RMSD (Å)	GH Family/Activity Associated	Templates (PDB ID)	Structural Analogs (PDB ID)
k141_2995_1_516_-	S461623	0.39	0.77 ± 0.10	4.2 ± 2.8	GH10/ xylanase	1vbr, 6fhe, 5ofj, 3emq, 2q8x, 2uwf, 3u7b	5ofj, 3ms8, 2uwf, 3emc, 1r85, 2f8q, 1clx, 4w8l, 2cnc, 2dpe
k141_6861_1_322_+	S461626	0.24	0.75 ± 0.11	3.6 ± 2.5	GH43/ arabinofuranosidase	4qqs, 3kst, 1yrz, 4kca, 4kc7, 5ho0	4qqs, 3kst, 5z5d, 1yi7, 5jow, 1yrz, 3qz4, 1yif
k141_9036_1_604_-	S461628	0.99	0.85 ± 0.08	3.4 ± 2.4	GH3/ endo-glycosidase	5vqd, 5vqe, 3cqm, 1tr9, 3bmx, 5bu9, 3sql	3nvd, 5qve, 3sqm, 5jp0, 4zm6, 5yot, 5m6g, 3f93, 1iew, 5bu9
k141_10879_1_560_-	S461631	0.12	0.73 ± 0.11	4.9 ± 3.2	GH5/ endo-1,4-β-mannanase	1rh9, 4lpy	1rh9, 3pzm, 4l3m, 1uuq, 4lpy, 3wh9, 1pnp, 3ziz, 4awe, 2xg9
k141_9497_1_728_+	S461630	0.22	0.74 ± 0.11	5.3 ± 3.4	GH30/ endo-β-1,6-glucanase xylanase	3clw, 2v3e, 5ngk, 2wnw	3clw, 2f61, 2wnw, 5ngk, 6iuj, 4b3k, 3kl3, 5ndx, 5bx9, 5ta0
k141_15375_1_491_-	S461835	0.62	0.80 ± 0.09	3.7 ± 2.5	GH5/ endo-1,4-β-mannanase	3pz9, 4l3m, 3wfl, 1rh9, 4lyp	3pzm, 4l3m, 1rh9, 3wh9, 3wfl, 1qno, 3ziz, 4awe, 1uz4, 4lyp
k141_14357_218_1033_-	S461832	0.22	0.74 ± 0.11	5.5 ± 3.5	GH15/ endo-β-1,2-glucanase glucoamilase α-1, 2-mannosidase	5gzh, 5z06, 4gl3	5gzh, 5z06, 4gl3, 3eu8, 6imu, 6fhw, 2vn4, 1gly, 1dl2
k141_11065_1_594_-	S461829	1.30	0.89 ± 0.07	2.8 ± 2.0	GH3/ endoglucanase β-xylosidase	5yot, 5z9s, 5jp0, 5z87	5z87, 5yot, 5jp0, 5z9s, 5a7m, 4zo6, 1ex1, 3u48, 5m6g, 3ut0
k141_3756_1_1250_-	S461624	0.92	0.60 ± 0.14	9.0 ± 4.6	GH10/ endo-β-1,4-xylanase	1isy, 2cnc, 4l4o, lisv, 4k68, 2q8x, 1vbr	1isy, 4w8l, 1us2, 2cnc, 5ofj, 3ms8, 3emz, 4l4o, 4k68
k141_827_1470_3344_-	S461622	0.03	0.71 ± 0.12	7.9 ± 4.4	GH55/ β-1,3-glucanase	5m5z, 3eqn	3eqn, 5m5z, 4pew, 5gkd, 5ggc, 1ofl, 1rmg, 5z9t, 4oj5, 3zzp
k141_13601_1_1245_-	S461831	0.81	0.61 ± 0.14	8.7 ± 6.4	G51/ arabinofuranosidase glucanase-xylanase	6d25, 2vrq	6d25, 2vrq, 2c8n, 2y2w, 1pz3, 3ug4, 3vny, 5bwi, 3ii1, 2j25
k141_19777_314_775_-	S462092	0.44	0.77 ± 0.10	3.9 ± 2.7	GH5/ endo-1,4-β-mannanase	4l3m, 1rh9, 3wh9	4l3m, 3pzm, 1rh9, 4awe, 3wh9, 3wfl, 3ziz, 1qnp, 1uuq, 4lyp
k141_35613_1609_2534_-	S466607	0.63	0.80 ± 0.09	4.9 ± 3.2	GH30/ β-glucosidase	3clw, 6iuj, 2v3e,	3clw, 6iuj, 2c8n, 1pz3, 2f61, 3kl0, 4qaw, 5cxp, 6d25, 4fmv
k141_39467_1_958_-	S466608	0.18	0.74 ± 0.11	5.9 ± 3.7	GH10/ endoxylanase	1xyz, 5ofj, 3wub, 4k68, 2q8x, 2uwf	1hiz, 6fhf, 2fgl, 2uwf, 5ofj, 3msd, 3emc, 4l4o, 1us3, 4w81
k141_40844_1_1278_+	S466609	0.83	0.83 ± 0.08	5.2 ± 3.3	GH51/ α-N-arabinofuranosidase	6d25, 2vrq	6d25, 2vrq, 1p23, 2c8n, 2y2w, 3s2c, 3vo0, 3ik2, 2yjg, 3fw6
k141_42666_1_1020_-	S466610	1.27	0.89 ± 0.07	3.9 ± 2.6	GH3/ β-N-acetylglucosaminidase	3sq1, 3cqm, 4zm6, 5vqd	3sql, 3sqm, 3nvd, 5vqd, 4zm6, 5jp0, 5m6g, 1iew, 5yot, 5bu9
k141_46724_1_745_+	S466611	0.37	0.76 ± 0.10	5.0 ± 3.2	GH5/ endo-1,4-β-mannanase	1rh9, 1uuq, 4lpy, 4l3m	1rh9, 4l3m, 3pzg, 1uz4, 3wh9, 4awe, 3wfl, 1qno, 3ziz, 4lyp

## Supplementary Materials

Supplementary materials can be found at https://www.mdpi.com/2076-2607/7/12/619/s1. Table S1. Metagenomic data used in the comparison. Figure S1. Taxonomic assignment of Archaea (upper) and Bacteria (lower) in halite metagenome. Figure S2. Biophysical properties of the predicted proteomes across Kingdoms in the metagenomic sequences from Atacama Desert halites. Figure S3. Pathway enrichment analysis related to photosynthesis. Red boxes represent enzymes found in the halite´s metagenome. Figure S4. Pathway enrichment analysis related to Carbon fixation mechanisms in photosynthetic organisms. Figure S5. Pathway enrichment analysis related to Carbon fixation mechanisms in prokaryotes. Red boxes represent enzymes found in the halite´s metagenome. Figure S6. Pathway enrichment analysis related to Nitrogen metabolism. Figure S7. Pathway enrichment analysis related to Sulfur metabolism. Figure S8. (A) Location of Salar Grande at Atacama Desert, Chile. (B) Panoramic view of halites in Salar Grande. Halite rocks are marked with black arrows. (C) Fractured halite rocks. Green zone represents endolithic microbial colonization. (D–F) Different views of Atacama Desert halite rocks.
