# Supplementary material for: Metagenomics of Atacama Lithobiontic Extremophile Life Unveils Highlights on Fungal Communities, Biogeochemical Cycles and Carbohydrate-Active Enzymes
Source: Microorganisms. 2019 Nov 27;7(12):619. doi: 10.3390/microorganisms7120619 (PMC6956184; doi:10.3390/microorganisms7120619)
Supplement: Supplementary file 1 [file microorganisms-07-00619-s001.pdf]

Table S1. Metagenomic data used in the comparison.

Sample ID	SRA Number	Sequencing Platform	Type of Library	Country	Coordinates (Sampling site)	Collection Data	Reference
Crust 1	SRR6651926	Illumina HiSeq 2000	Paired end librery	Chile	21.21, 69.84	June 2013	Finstad *et al.* (2017)
Crust 2	SRR6651936						
Crust 3	SRR6651949						
Endolithic 1	SS2630167	Illumina HiSeq 2000	Paired end librery	Chile	20.93, 70.01	April 2013	Crits-Christoph *et al.* (2016)
Hypolithic	SRR212483	Illumina HiSeq 2000	Paired end librery	Namibia	23.32, 15.01	April 2010	Vikram *et al.* (2016)
Desert soil 1	ERR1353018	Illumina HiSeq 2500	Paired end librery	USA	33.28, -116.13	2012	N.R.
Desert soil 2	ERR1352897	Illumina MiSeq		Spain	400, 37	2015	
Desert soil 3	ERR1352898						
Desert soil 4	ERR1352472	Illumina HiSeq 2500		USA	33.28, -116.13	2012	
Hypersaline 1	ERR1739732	Illumina HiSeq 4000	Paired end librery	Iran	34.18, 49.83	November 2013	Naghoni *et al.* (2017)
Hypersaline 2	ERR1739731						
Hypersaline 3	ERR1743002						
Hypersaline 4	ERR1739733						
Hypersaline 5	ERR1742998						

N.R.: No reference is available

References:

Crits-Christoph, A., Gelsinger, D.R., Ma, B., Wierzchos, J., Ravel, J., Davila, A., Casero, M.C., DiRuggiero, J. Functional interactions of archaea, bacteria and viruses in a hypersaline endolithic community. *Environ. Microb*. **2016**, *18*, 2064-2077.

Finstad, K.M., Probst, A.J., Thomas, B.C., Andersen, G.L., Demergasso, C., Echeverría, A., Amundson, R.G., Banfield. J.F. Microbial community structure and the persistence of cyanobacterial populations in salt crusts of the hyperarid Atacama Desert from genome-resolved metagenomics. *Front. Microb*. **2017**, *8*, 01435.

Naghoni, A., Emtiazi, G., Amoozegar, M.A., Cretoiu, M.S., Stal, L.J., Etemadifar Z., Fazeli, S.A., Bolhuis, H. Microbial diversity in the hypersaline Lake Meyghan, Iran. *Sci. Rep*. **2017**, *7*, 11522.

Vikram, S., Guerrero, L.D., Makhalanyane, T.P., Le, P.T., Seely, M., Cowan, D.A. Metagenomic analysis provides insights into functional capacity in a hyperarid desert soil niche community. *Environ. Microb*. **2016**, *18*, 1875-1888.

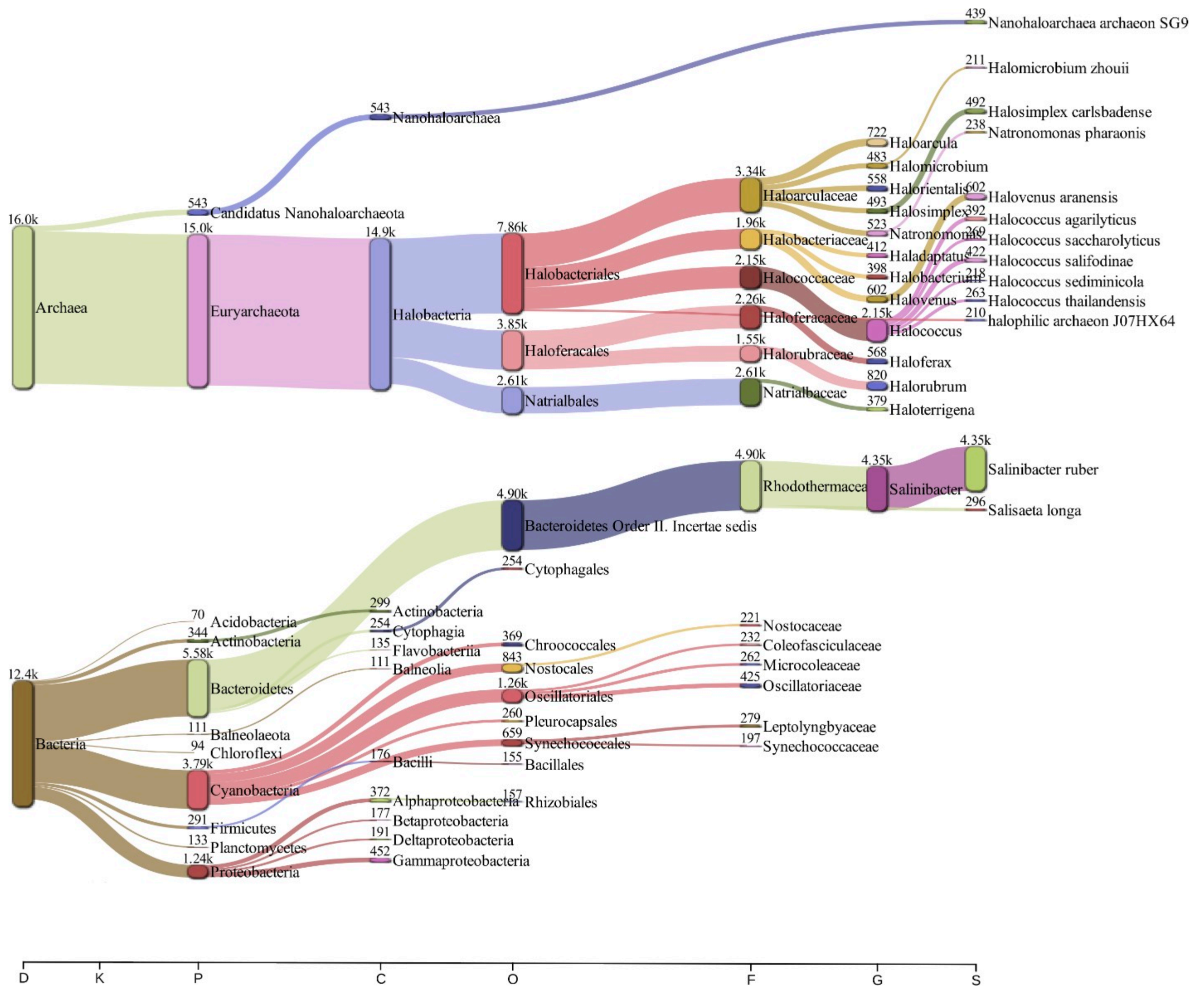

Figure S1. Taxonomic assignment of Archaea (upper) and Bacteria (lower) in halite metagenome. Horizontal axis: D – Domain, K – Kingdom, P – Phylum, C – Class, O – Order, F – Family, G – Genus, S – Species.

A

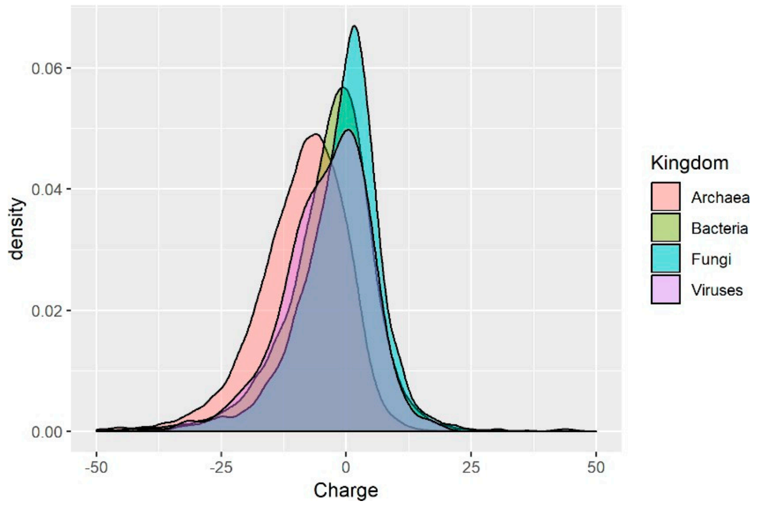

B

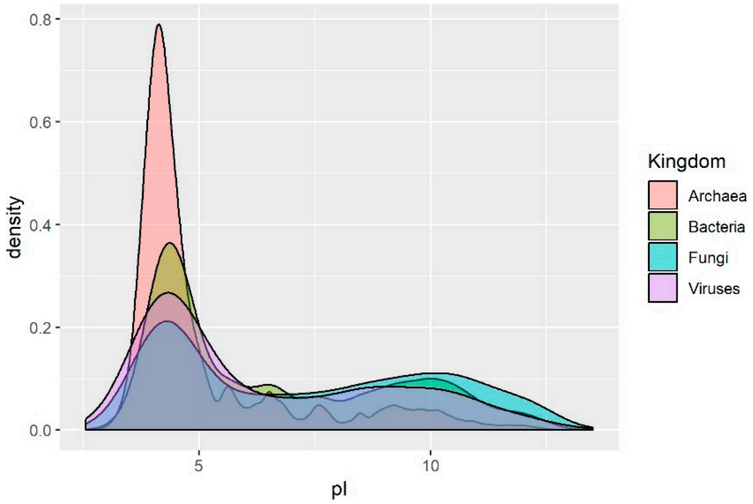

C

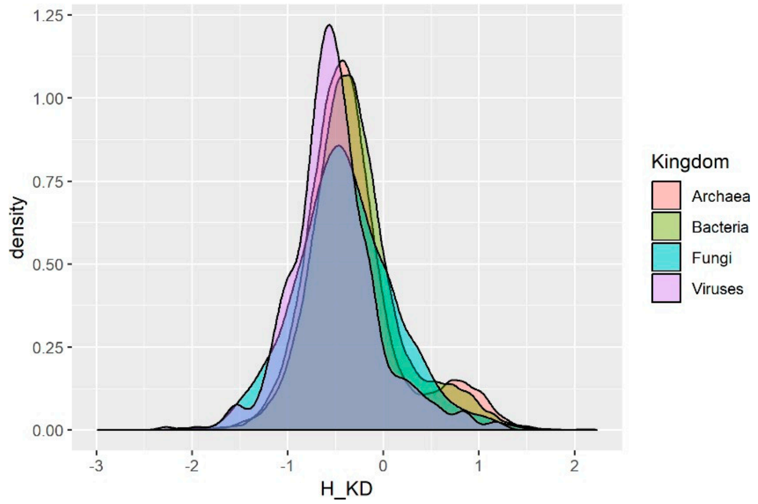

Figure S2. Biophysical properties of the predicted proteomes across Kingdoms in the metagenomic sequences from Atacama Desert halites. (**A**) Charge in total proteome, (**B**) pI in total proteome, (**C**) Kyte-Doolittle Hydrophobicity (H_KD) index in total proteome.

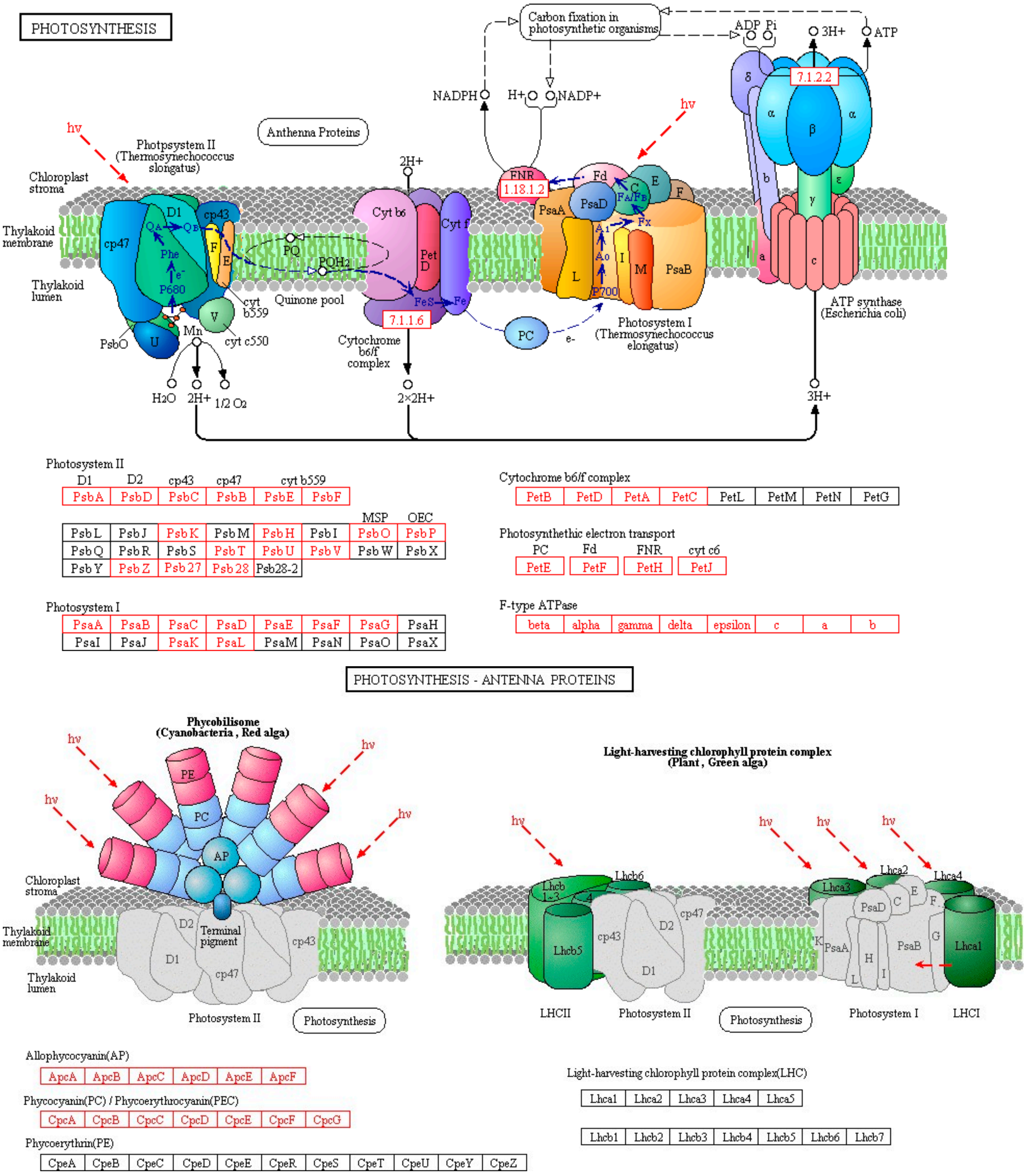

Figure S3. Pathway enrichment analysis related to photosynthesis. Red boxes represent enzymes found in the halite's metagenome.

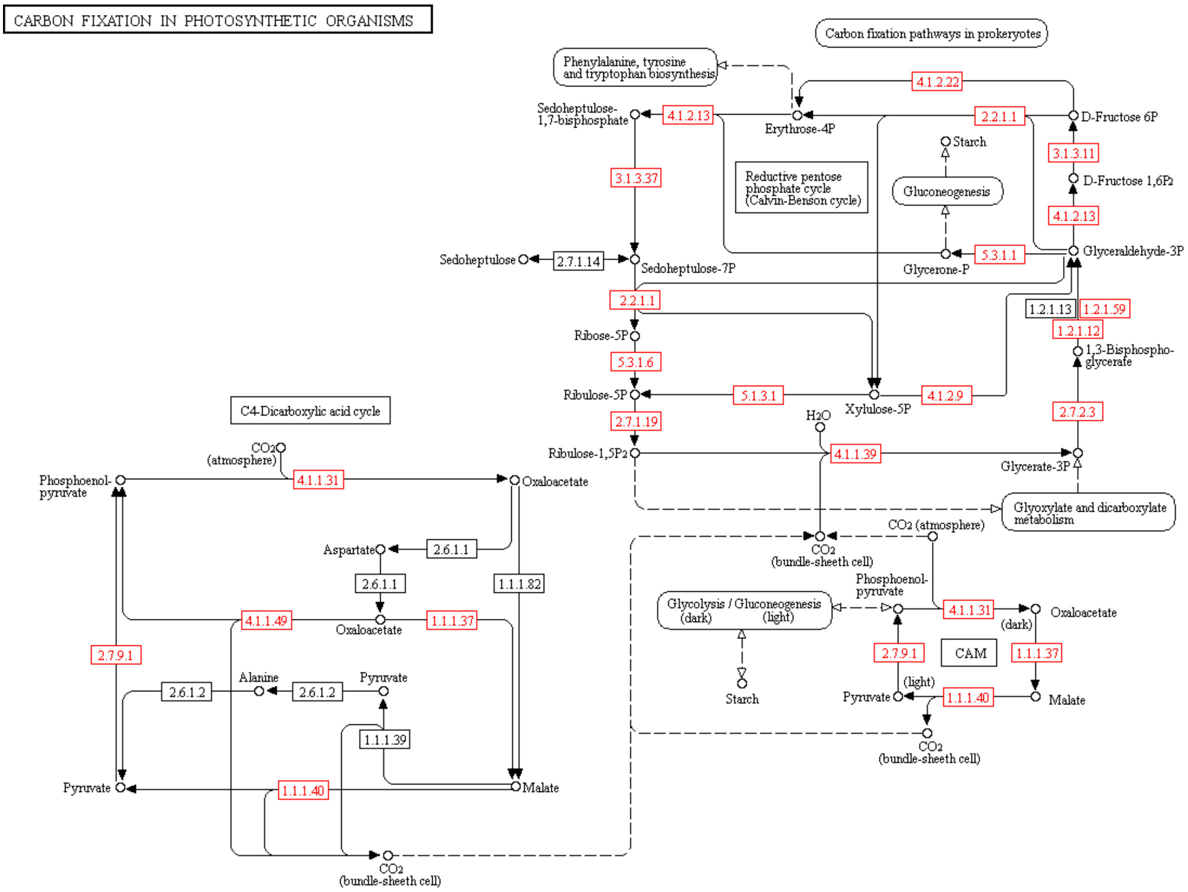

Figure S4. Pathway enrichment analysis related to Carbon fixation mechanisms in photosynthetic organisms. Red boxes represent enzymes found in the halite's metagenome.

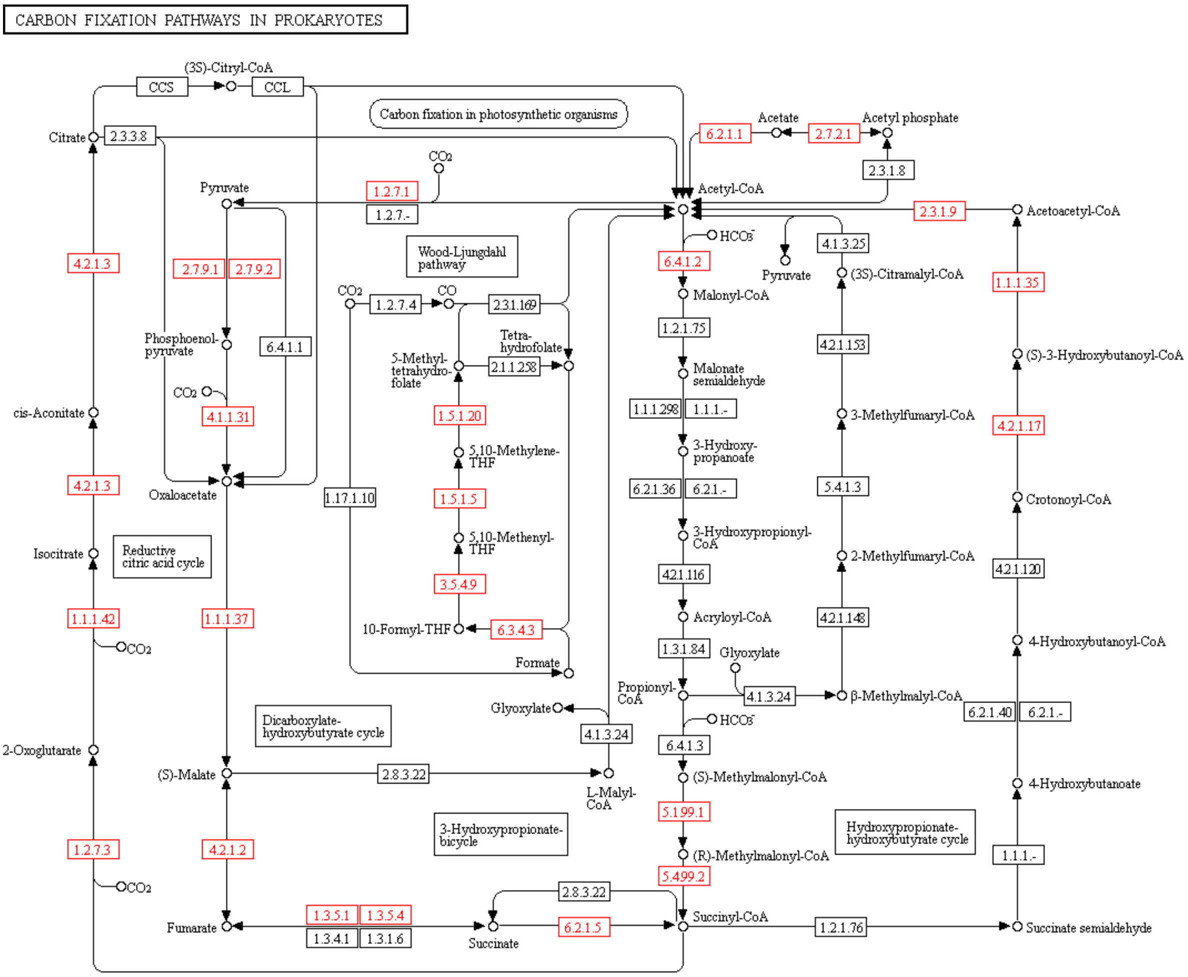

Figure S5. Pathway enrichment analysis related to Carbon fixation mechanisms in prokaryotes. Red boxes represent enzymes found in the halite's metagenome.

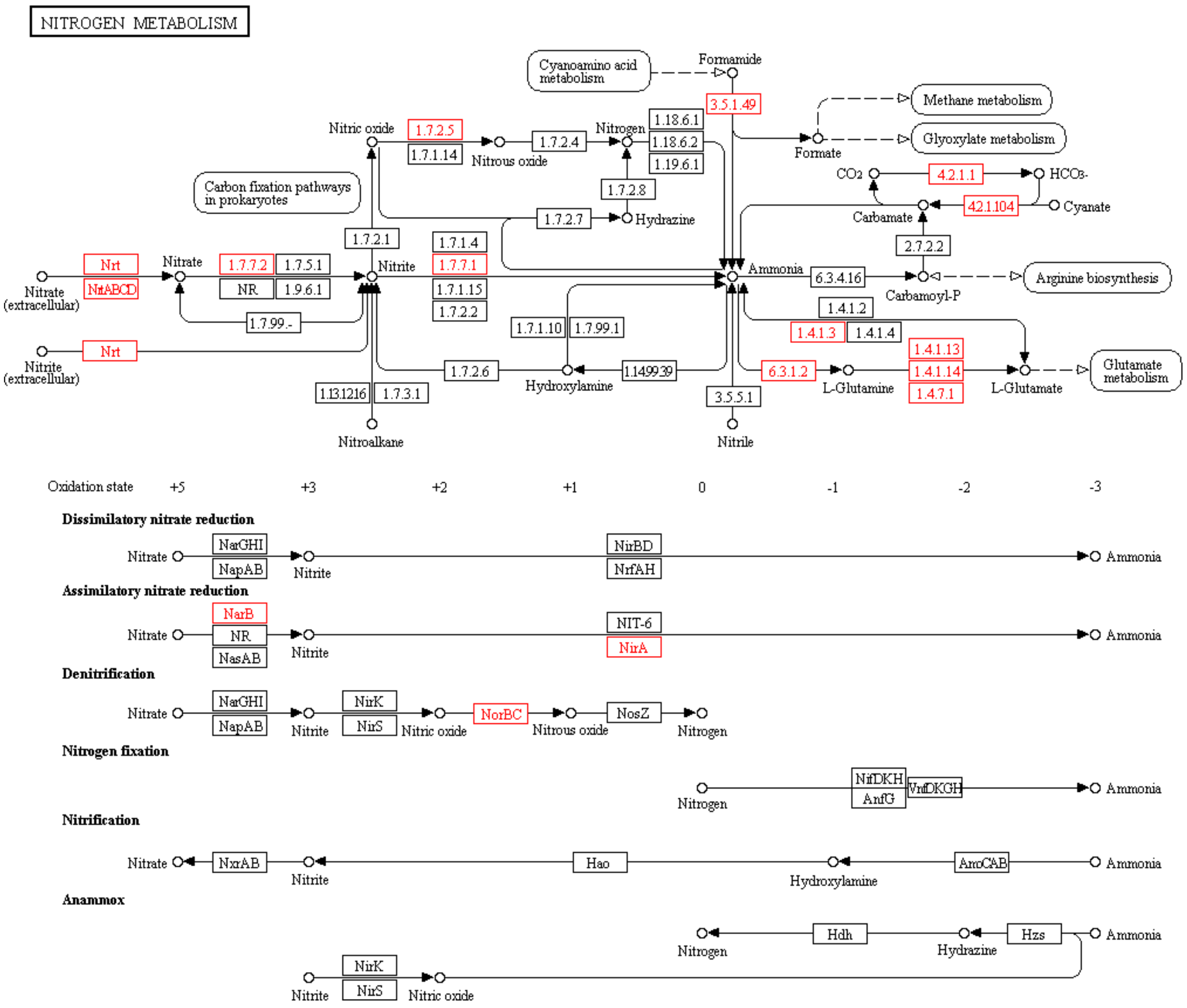

Figure S6. Pathway enrichment analysis related to Nitrogen metabolism. Red boxes represent enzymes found in the halite's metagenome.

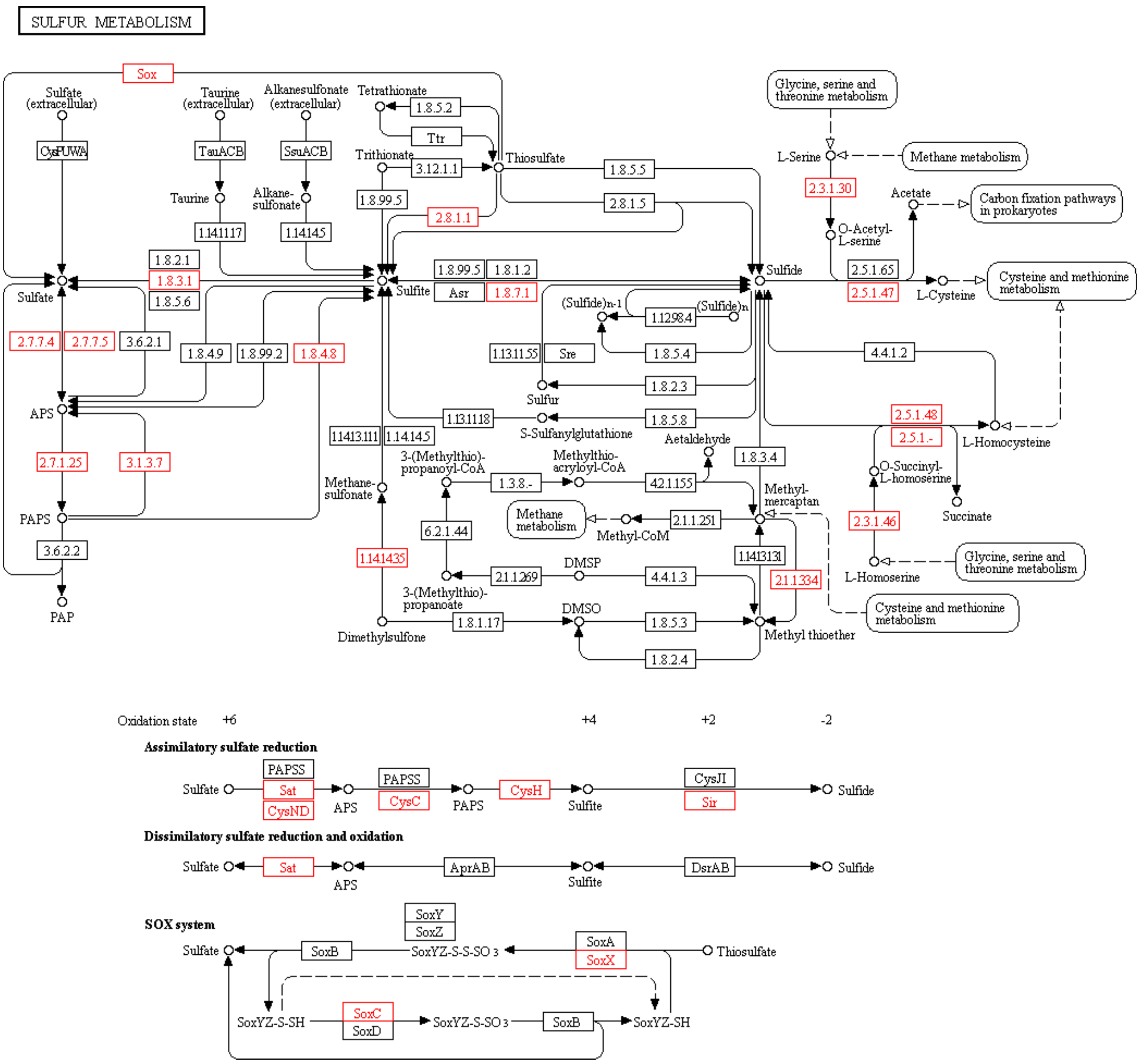

Figure S7. Pathway enrichment analysis related to Sulfur metabolism. Red boxes represent enzymes found in the halite's metagenome.

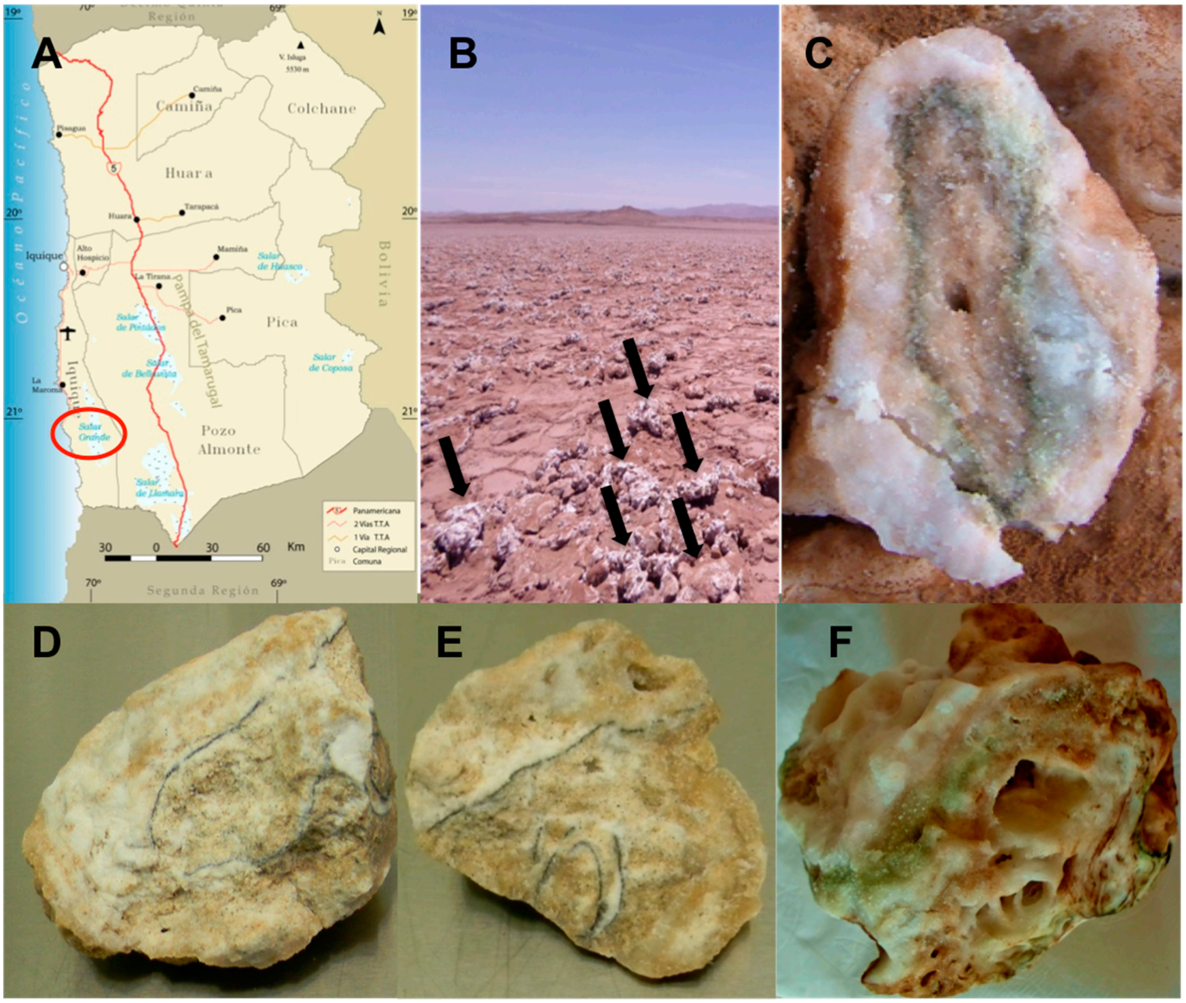

Figure S8. (A) Location of Salar Grande at Atacama Desert, Chile. (B) Panoramic view of halites in Salar Grande. Halites rocks are marked with black arrows. (C) Fractured halite rock. Green zone represents endolithic microbial colonization. (D, E and F) Different views of Atacama Desert halite rocks.
